# Numerical assessment of optoelectrical properties of ZnSe–CdSe solar cell-based with ZnO antireflection coating layer

**DOI:** 10.1038/s41598-023-38906-z

**Published:** 2023-07-27

**Authors:** D. Parajuli, Devendra KC, Khim B. Khattri, Dipak Raj Adhikari, Raid Anam Gaib, Deb Kumar Shah

**Affiliations:** 1 Research Center for Applied Science and Technology, Tribhuvan University, Kirtipur, 44613 Nepal; 2 Lebesby Kommune, Norway; 3 Department of Mathematics, School of Science, Kathmandu University, Dhulikhel, 45210 Kavre Nepal; 4 Graduate School of Science and Technology, Mid-west University, Birendranagar, 21700 Nepal; 5 Department of Electrical and Electronic Engineering, University of Tromso, 9019 Tromso, Norway; 6 School of Semiconductor and Chemical Engineering, Jeonbuk National University, Jeonju, 54896 Republic of Korea

**Keywords:** Energy science and technology, Materials science, Optics and photonics, Physics

## Abstract

In this work, a numerical assessment of the optoelectrical properties of the ZnO–ZnSe–CdSe heterojunction for a thin and cost-effective solar cell was made by using the PC1D simulation software. The photovoltaic (PV) properties have been optimized by varying thicknesses of the absorber layer of the p-CdSe layer, the window layer of n-ZnSe, and the antireflection coating (ARC) layer of ZnO, a transparent conductive oxide with enhanced light trapping, and wide bandgap engineering. There is a positive conduction band offset (CBO) of ΔEc = 0.25 eV and a negative valence band offset (VBO) of ΔEv = 1.2 − 2.16 =  − 0.96 eV. The positive CBO prevents the flow of electrons from the CdSe to the ZnSe layer. Further, the impact of doping concentration on the performance of solar cells has been analyzed. The simulation results reveal the increase in the efficiency of solar cells by adding an ARC. The rapid and sharp increase in the efficiency with the thickness of the window layer beyond 80 nm is interesting, unusual, and unconventional due to the combined effect of morphology and electronics on a macro-to-micro scale. The thin-film solar cell with the structure of ZnO/ZnSe/CdSe exhibited a high efficiency of 11.98% with short-circuit current (I_sc_) = 1.72 A, open-circuit voltage (V_oc_) = 0.81 V and fill factor (FF) = 90.8% at an optimized thickness of 2 μm absorber layer, 50 nm window layer, and 78 nm ARC layer. The EQE of solar cells has been observed at about 90% at a particular wavelength at 470 nm (visible light range). Around 12% of efficiency from such a thin-layered solar cell is highly applicable.

## Introduction

With the gradual decline of nonrenewable energy sources such as petroleum, coal, and natural gas, clean or renewable energy, has emerged as humanity's inevitable savior^1–3^. Solar energy is an excellent option for green and sustainable resources that will facilitate resolving the enormous problems of the energy crisis and environmental concerns^4^**.** The design of a solar cell is done in such a way that there is optical band alignment^5^ at the heterojunction for the overall device efficiency, stability, and scalability. Similarly, interface engineering and doping concentration play their role to enhance carrier transport and limit recombination losses. Likewise, the quality of material and impurities, light absorption, and photon management affect greatly the solar cell system. On the other hand, the cost of the material used matters a lot in scale production. So, consequent investigations on cost-effective production with respect to the cost and the amount of material used were discussed since 1982^6^, are in focus. Fabrication of solar cells with a thinner antireflecting coating (ARC), window, and absorber layer is one of the approaches that significantly help in this regard.

Currently, II–VI semiconductor compounds (such as CdSe, ZnSe, and ZnTe) with higher stability and tenability are considered promising materials with higher photovoltaic performance^7^**.** ZnSe itself is a much more promising material for window layer^8^ with high efficiency and low cost. CdSe and ZnSe both have a higher capacity for photon absorption in the visible region of wavelength 400–750 nm^9^**.** CdSe has very similar characteristics to CdTe and CdSe has also a direct bandgap semiconductor that has a high absorption coefficient (α = 10^4^ cm^−1^ at 720 nm)^10^. Therefore, the CdSe solar cell needs only a very thin (~ 2 µm) film to absorb the sunlight for higher power conversion efficiency (PCE). The ZnSe is a non-toxic material as compared to CdSe and has a higher conduction band edge^11^**.** In addition, ZnSe material is photosensitive with a wider large direct band gap appropriate for LEDs and lasers^12,13^ with a wider transparency range for the window layer of solar cells ^14^. Though it is highly efficient for solar cells, cadmium is a toxic heavy metal^15^ upon long-term exposure to the environment which has to be prevented from contamination. The CdSe have limited stability in the presence of moisture and oxygen whose degradation affect the performance and lifetime of the solar cells^16^. So, its encapsulation or protective coating is also necessary besides its cost and complexity in preparation. Similarly, band offset between ZnSe and CdSe may lose the photogenerated carriers by carrier recombination^17^. The scale production might be challenging for fabrication which needs a high-quality deposition technique with precise control from layer to layer. Several studies have been performed on the CdSe absorber layer-based solar cell such as the changes in the TiO_2_ photoanode's structure in CdS/CdSe-sensitized solar cells were studied and forwarded the double-layer photoanode with 4.92% PCE at 0.15 cm^2^ photoactive area^18^. KC et al. has optimized the ZnSe window layer combined with the absorber layer for GaAs solar cells^8^. Frese et al. has presented CdSe photoelectrochemical solar cell with a conversion efficiency of 12.4% in alkaline K_3_Fe(CN)_6_/K_4_Fe(CN)_6_ electrolyte^19^**.** Aghmiyoni et al. have used pentacene-doped PEDOT: PSS layer for the injection of holes and their optoelectrical simulations have been studied of P_3_HT: CdSe hybrid solar cell performance. It was found that the work function of the layer has been reduced from 5.1 to 4.9 eV as a result the efficiency was improved^7^**.** Dey et al. applied an AMPS-1D simulator along with absorber CdSe and the n-type-ZnS buffer layer and analyzed the thickness of the layer, doping concentration, and temperature. The ITO/ZnS/CdSe structure with a 1.2 μm thick absorber exhibited the PCE = 17.35%, J_sc_ = 13.82 mA/cm^2^, V_oc_ = 1.38 V, and FF = 0.908^20^**.** Similarly, Monika et al. studied the efficiency of CdS solar cells after sensitization and passivation. The heterojunctions of type II with TiO_2_-CdS-CdSe exhibited the transfer of electrons to the anode is doubled which enhances the PCE^21^. Abdalameer et al. prepared the ZnSe nanoparticles using Zinc metal sheets and selenium nitrate and its core cell with the plasma jet system for the window layer of the solar cell and the resultant n-ZnSe/p-Si was found to be with the efficiency tuning from 0.89 to 2% with the porous time (5–20 min)^22^.

With an expectation of limiting the above environmental along with other several efficiency limiting problems, the antireflecting coating (ARC) technique is preferred. Maximum absorption of the solar radiation on the solar cell is the major challenge that can be employed by the use of an ARC layer like ZnO, SiN_x_, MgO, TiO_2_, Al_2_O_3_, ZnS, etc.^23^. Among them, ZnO is more efficient due to its wide band gap (3.44 eV at low temperature and 3.37 eV at room temperature) giving wider transparency in the visible and near ultraviolet range of the spectrum^24^. ZnO can be easily prepared oxide for its use in diverse applications due to its wider bandgap and good anti-reflection capabilities^25^. ZnO coatings can also act as light-trapping structures, improving light absorption inside the solar cell. ZnO surface has the ability to scatter light, lengthening photons paths inside cells and encouraging absorption. This light-trapping effect is especially advantageous for thin-film solar cells, where a higher absorption of light can make up for the thinner active layers^26^. ZnO coatings have strong mechanical and chemical stability, increasing their durability and resistance to the effects of the environment^27^. There are other ARC coatings like TiO2 and MgF2 under investigation, but their capacity to minimize reflection over a broad wavelength range was constrained^28^. They were not restive to moisture, UV light, or other extreme conditions that cause their coatings to be degraded resulting in a reduction in their anti-reflection qualities and general performance in solar cells^29^. The band gap of ZnO is ~ 3.37 eV, MgF2 (Magnesium Flouride) is ~ 7.8 eV and that of TiO2 (Anatase & Rutile) is ~ 3.0–3.2 eV. Flores et al. in their computational bandgap alignment study reported that the ZnO/ZnSe with ~ 1.71 eV core–shell band gap^30^ exhibiting type-II bandgap alignment^31^ is good for photovoltaic devices.

Using ZnO in combination with ZnSe–CdSe seems to be specific and novel. The bandgap energy of ZnSe–CdSe and their allied optical properties may make a difference in the performance of the solar cell. In addition, the ZnO–ZnSe–CdSe combination shows photoelectrochemical performance for the in-anion exchange phenomenon^32^. The ZnO-coated solar cell also shows a maximum of 28.04% of PCE CdS/CdTe based solar cell as reported by Ahmmed et al.^17^ As a result, the new combination will be a single-layered ARC (SLARC) ZnO material for the ZnSe and CdSe solar cells to enhance the efficiency of the proposed solar cell. The preliminary investigation determines its further study with respect to long-term stability and reliability.

In this work, the PC1D simulation tool was used for the study of the optoelectronic properties of ZnO–ZnSe–CdSe solar cells in which the photovoltaic parameters were analyzed with the variation of thickness of the absorber, window, and antireflection layers through their optimization.

## Device architecture and simulation tool

**Figure 1:**
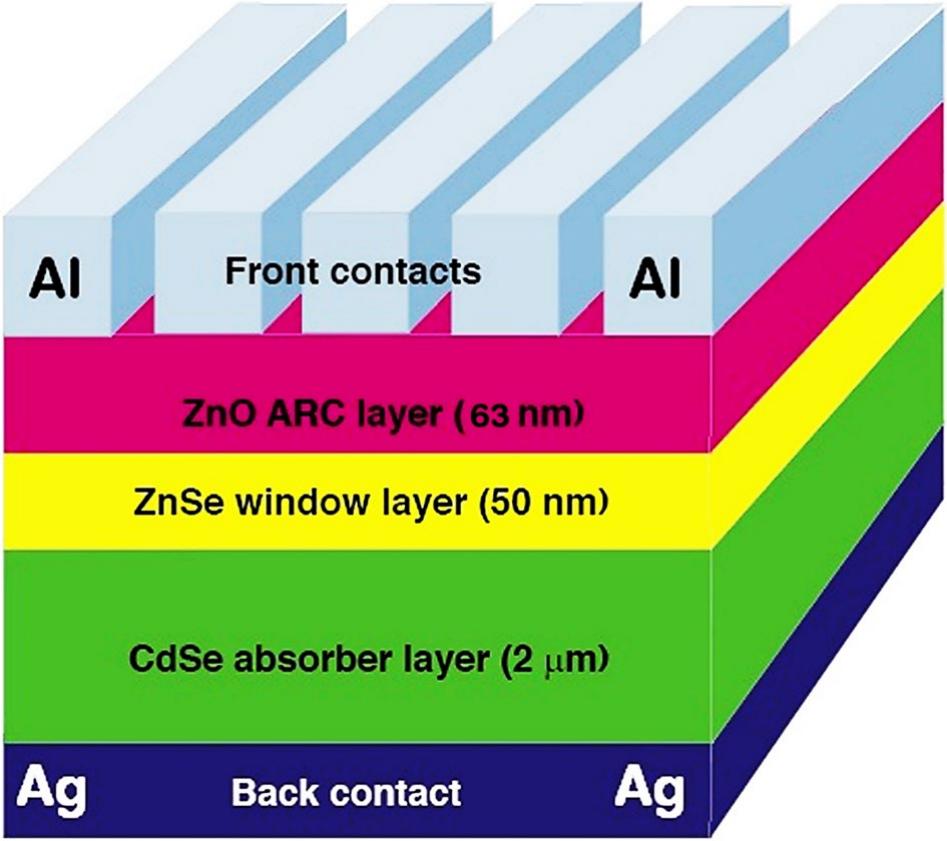
Schematic of ZnSe/CdSe solar cell with ZnO as ARC.

The device architecture of the purposed solar cell is as in Fig. 1. In this schematic structure, CdSe has been chosen as an absorber layer and acts as p-type material. Similarly, ZnSe has been chosen as a window layer, which acts as n-type material lying between the ZnO ARC layer and p-type material with a device area of 100 cm^2^ respectively. The electrode materials of most of the semiconductors are good due to their low VBO i.e. low valence barrier and electron reflecting ability, and higher CBO. The appropriate choice of material for back contact will improve the short circuit current limit of the CdSe layer^33^. However, we have used Silver (Ag) as the back contact electrode and Aluminium (Al) as the front contact electrode as we have incorporated it in our previous work^34^.

There are various simulation software for solar cells, among them, PC1D simulation has been chosen because of free availability, open source, and long publication history^35^**.** The PC1D tool is used to simulate the optoelectrical properties of the ZnSe–CdSe Solar cell with inset parameters listed in Table 1^36–41^. The standard solar radiation and light intensities are AM 1.5 and 0.1 W/cm^2^ (one sun) at 25 °C temperature.

**Table 1:** Inset parameters of the PC1D simulation tool.

Parameters	CdSe^20^	ZnSe^75^
Thickness	0.6–3 μm	30–100 nm
Energy bandgap (eV)	1.7	2.7
Electron Affinity	4.56	4.09
Bulk recombination	100 μs	100 μs
Doping concentration	1 × 10^16^–1 × 10^20^ cm^−3^	1 × 10^16^–1 × 10^20^ cm^−3^
Excitation mode	Transient	Transient
Electron mobility	650 (cm^2^/vs)	50 (cm^2^/vs)
Hole mobility	50 (cm^2^/vs)	20 (cm^2^/vs)
Constant Intensity	One sun	One sun
Dielectric constant	10.6	9.2
Temperature	300 K	300 K
Constant Intensity	0.1 W/cm^−2^	0.1 W/cm^−2^
Primary light source	AM 1.5 D spectrum	AM 1.5 D spectrum
Other parameters	Internal PC1D	Internal PC1D

## Result and discussions

### Band alignment and band offset

The band alignment (BA) and band offsets (BO)^42^ have a crucial role in the light reflectance, the transmission of photo charge carriers, and hence the efficiency of the solar cell^5,43^. There are three types of band alignment: (a) Type I (Straddling gap): Conduction band (CB) and valence band (VB) of the second is lower and higher than that of the first resulting in its band gap being narrower than that of first. (b) Type II (Staggered gap): Both the CB and VB of the second are lower than the first. (c) Type III (Broken gap): There is an overlapping of the CB of the second to the VB of the first resulting in the zero band gap between them.

**Figure 2:**
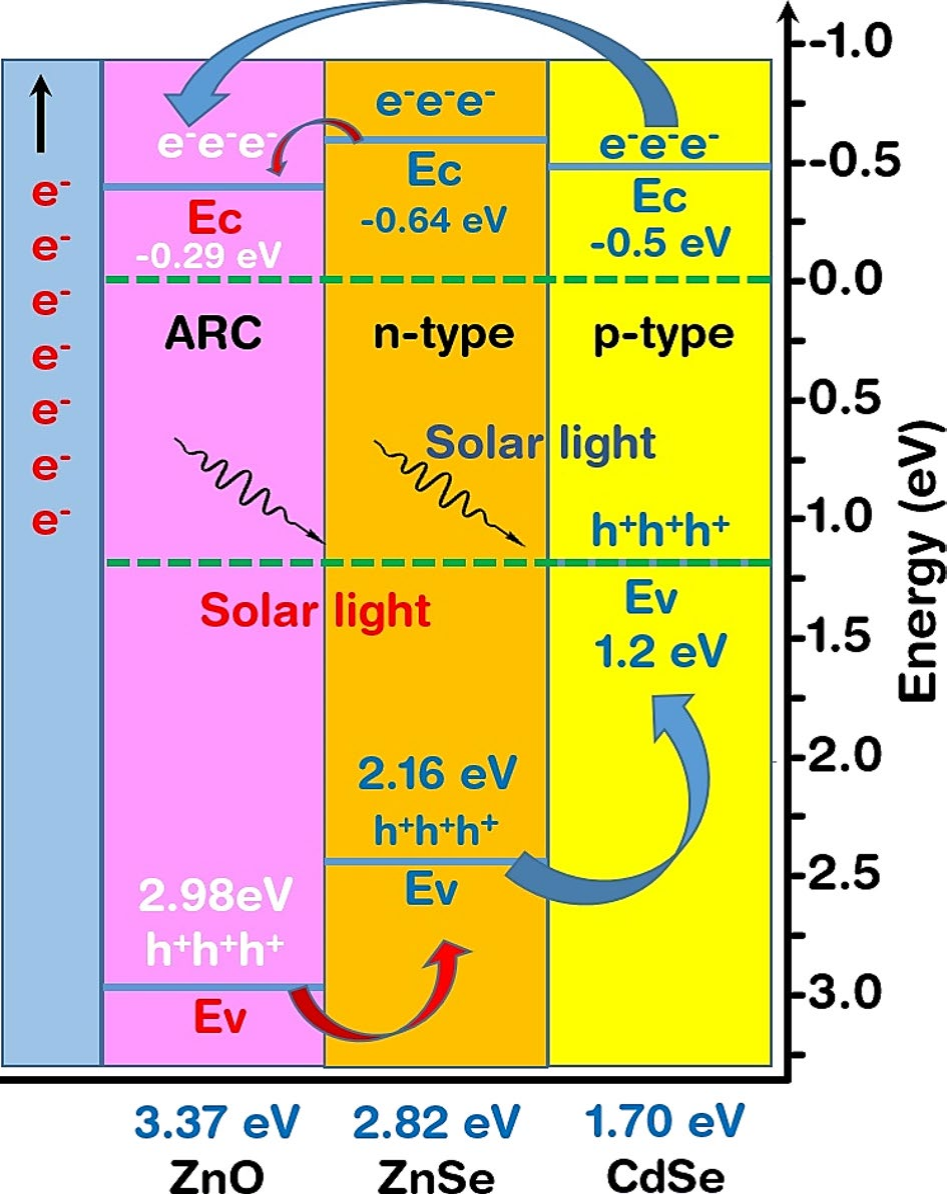
Band alignment and offset of the ZnO–ZnSe–CdSe solar cell.

The schematic band-alignment diagram of the ZnO–ZnSe–CdSe solar cell is shown in Fig. 2**.** Both the alignment is of type II showing the migration of photoelectrons from both junctions onto the ZnO. Here, the electrons are excited to the CB upon the incidence of photon on the substrate thereby creating a hole in the VB. Photo-generated charge carriers were separated under the illumination in semiconductors. The electrons excited by incident light jumped to the conduction band (CB), while the holes were left in the valence band (VB). The excited electrons are transferred from more to less negative potential in CB and the hole created is from more positive to less positive potential in VB^44^. Together with the electrons emitted by ZnO, they flow onto the external circuit. On the other end, the holes of ZnO in its VB migrate to the VB of ZnSe and then together to the VB of CdSe. This system has enhanced PEC due to the enormous light harvesting capacity of CdSe and the novel band structure of these combined heterojunctions with a capacity of transferring and separating the charges. This combination is in good agreement with the experimental work done by Lin & Wang^45^.

As the two energy bands of the semiconductors are aligned, interaction occurs, and a continuous Fermi level is maintained throughout the combination due to the discontinuous band structure. This relative alignment is band offset. The interface and bulk properties give the band offset and can be modified according to them^46^. Further, in the heterovalent junctions, the band offset is affected by geometry, orientations, interface bonds, and charge transfer between them^47^. The band discontinuity (difference in the bandgap of the valence band and conduction band) and built-in potential (the bands bend at the interface due to an imbalance of charges in the two semiconductors) gives the band offset following Poisson's equation.

Figure 2 shows the conduction band offset (CBO) and valence band offset (VBO) along with the electron affinity and band gap across the junction interface. The central or buffer layer has an energy gap of 2.82 eV and an electron affinity of 0.64 eV. It has positive BVO and negative CBO showing a reduction in carrier recombination. It has activation energy larger than that of the absorber^46,48^. The electron affinity of ZnSe (~ − 0.64 eV) is lower than that of CdSe (~ − 0.50 eV) indicating the possibility of positive CBO with ΔEc = 0.25 eV and negative VBO with ΔEv = 1.2–2.16 = − 0.96 eV. The positive CBO prevents the flow of electrons from the CdSe to the ZnSe layer. After the incidence of light on the surface of a material, a part of it is reflected which reduces the absorption and transmission of the photons whose energy depends on the bandgap of the material. If the energy of reflected photos coincides or is aligned with the material's conduction and valence band edges, the carrier concentration is increased. There is a transfer of electrons or holes thereby reducing the recombination process. So, the BO in the heterojunctions or interfaces between the different materials will reduce the transmission and increase the recombination process^42^. The BA or BO in the interface depends on the surface coating, interfacial layers, or doping which after optimization enhances transmission and reduces recombination for the higher efficiency of optoelectronic devices^49^.

### Impact of the thickness of absorber and window layers

**Figure 3:**
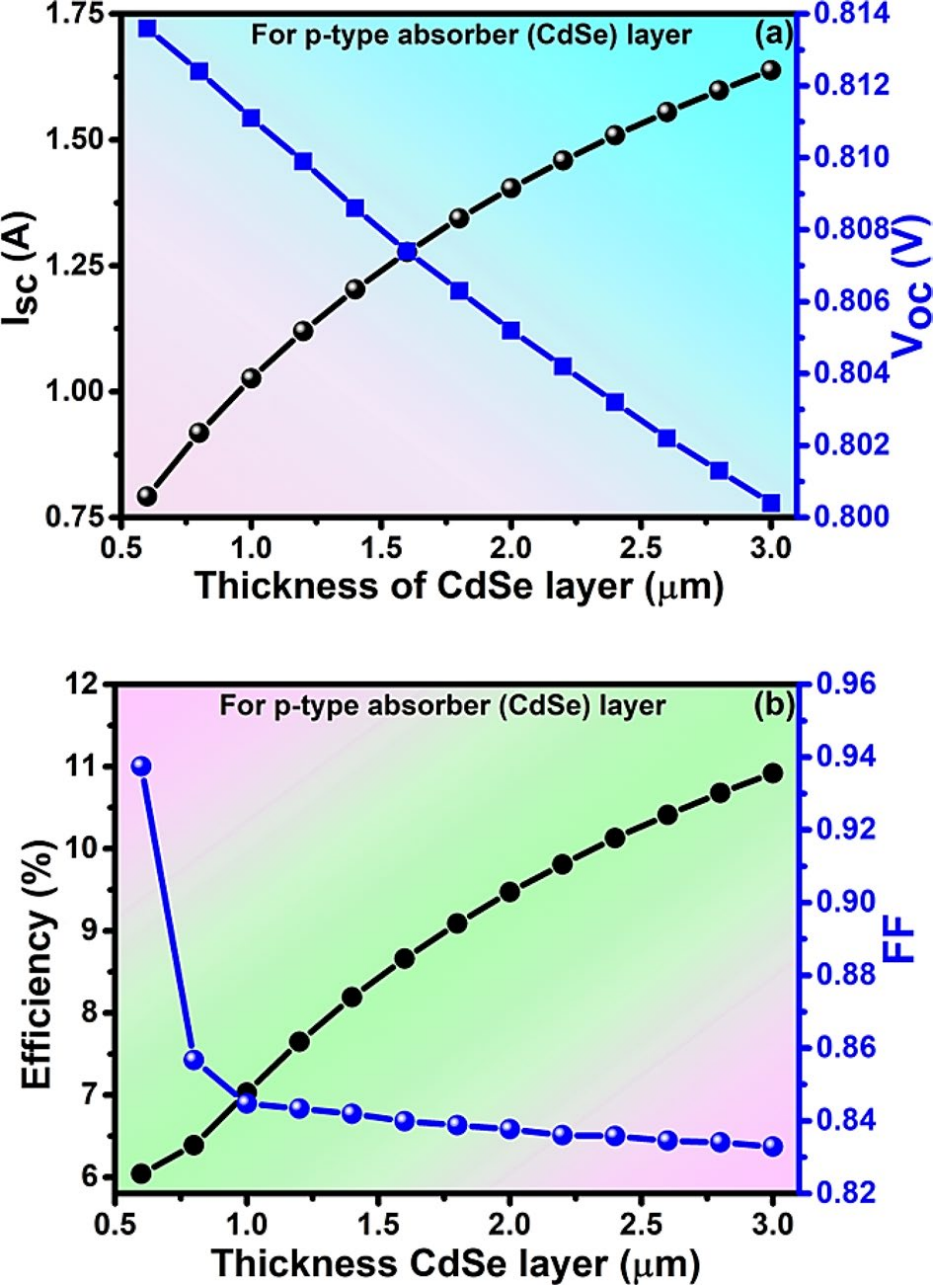
Analysis of (**a**) I_sc_ and V_oc_ (**b**) Efficiency and FF with the thickness of the absorber layer of solar cell.

The thickness of the absorber and window layer plays a crucial role in the solar cells' performance. As the thickness of the absorber layer increases, it traps more solar radiation thereby generating more charge carriers^50^**.** Whereas the window layer in combination with the absorber layer forms a p–n junction in a heterojunction thin-film solar cell to get a wider bandgap with smaller thickness and series resistance^51^. The thickness affects I_sc_, V_oc_, PCE & FF of the PV cell and is considered in the range of 0.5–3 µm for the absorber and from 10 to 100 nm for the window layer. The increases in absorber layer increase I_sc_ from 0.791 to 1.638 A as in Fig. 3a**.** It is due to more photons being absorbed thereby producing more electron pairs at the higher thickness and hence producing more photoelectric current^52^. The V_oc_ decreases from 0.813 to 0.800 V with an increase in the thickness of the absorber layer as in Fig. 3a due to more carrier recombination at higher thickness^19^. Similarly, the efficiency increases but the fill factor (FF) decreases with the thickness of the absorber layer due to more carrier recombination at higher thickness. The value of efficiency increases from 6.04 to 10.92% while FF decreases from 93.74 to 83.29% by varying the thickness of the absorber layer from 0.5 to 3 µm as in Fig. 3b. The optimized values of I_sc_ = 1.404 A, V_oc_ = 0.805 V, PCE = 9.47%, and FF = 83.76% have been observed at the optimized thickness of 2 µm of the absorber layer.

**Figure 4:**
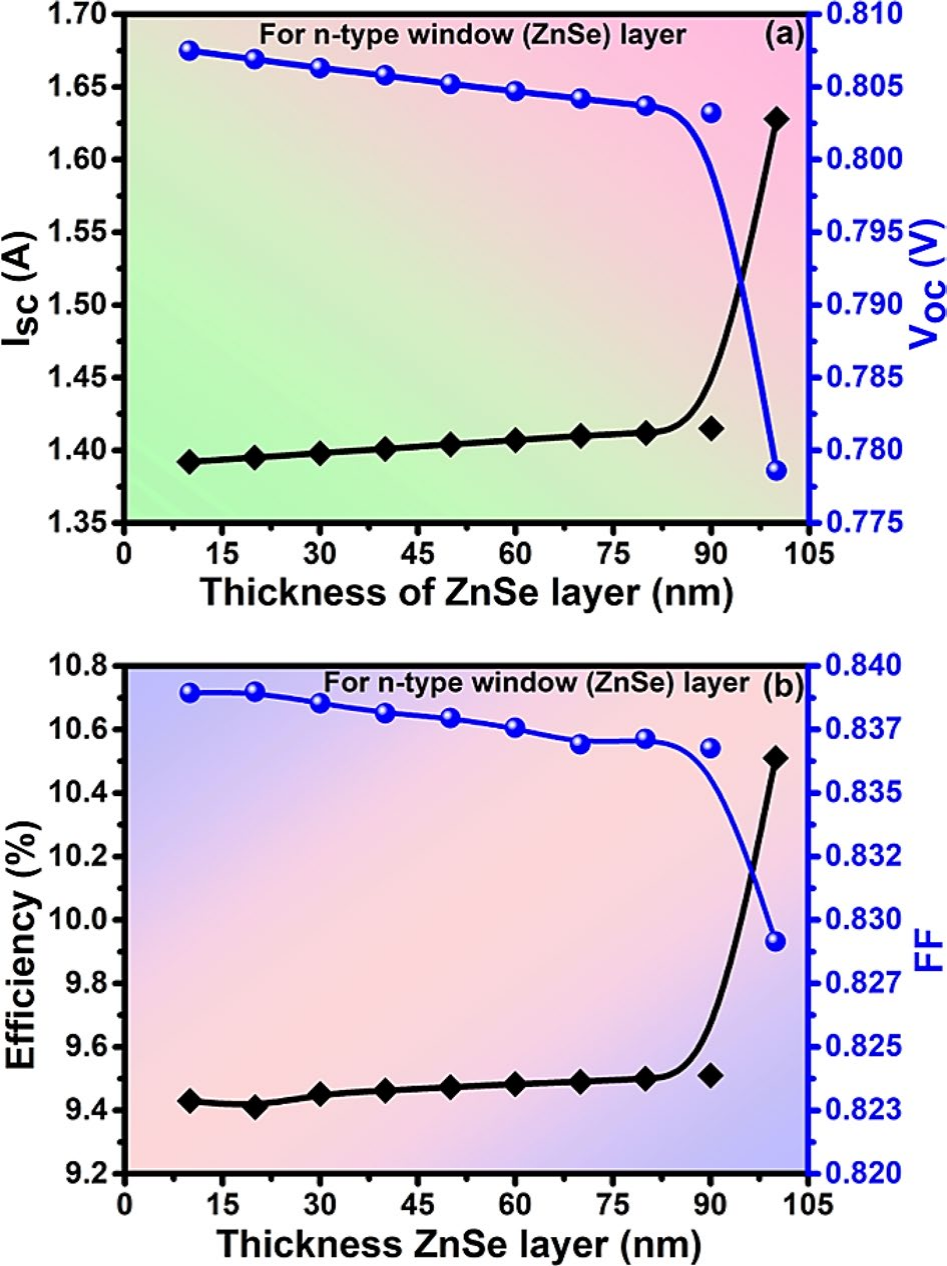
Analysis of (**a**) I_sc_ and V_oc_ (**b**)Efficiency and FF with the thickness of the window layer of solar cell.

Consequently, the value of I_sc_ increases from 1.392 to 1.628 A and the value of V_oc_ decreases from 0.807 to 0.778 V with an increase in the thickness of the window layer as in Fig. 4a. Also, the efficiency increase with increases in the window layer and FF decreases at higher thickness. The value of efficiency increases from 9.43 to 10.51% while FF decreases from 83.89 to 82.91% by varying the thickness of the window layer from 10 to 100 nm as in Fig. 4b. The optimized values of I_sc_ = 1.404 A, V_oc_ = 0.805 V, PCE = 9.473%, and FF = 83.79% have been observed at the optimized thickness of 50 nm of the window layer. The optimization of the thinner thickness of layers of materials of the solar cell helps to reduce the cost of fabrication.

Regarding the window layer of ZnSe, it minimizes the reflection loss by allowing the incident light toward the absorber layer. The layers are so optimized that they allow maximum photons through them. Similarly, the relatively thicker ZnSe layer, can absorb some percentage of the incident, and produce charge carriers that contribute to photocurrent generation which ultimately increases the current for better efficiency of the cell^53^. In order for achieving the thinner device structure as was done by Rickus 1982 with the material used, and the trade-off between transmission and absorption, the thickness of the window layer was taken to 50 nm. The relatively thicker single-layered ZnSe for higher photocurrent density leads toward the innovative application^6,54^. In Fig. 4b, the increasing efficiency with the thickness of the absorber without saturation may be due to consideration of range within the absorption limit. The saturation is reached only when the absorbing material does not contribute to fresh light absorption. Rather, it reabsorbs the already absorbed photons^55^. The recombination of the charge carriers, longer diffusion length, series resistance, and material quality may play a role in not reaching the system into saturation.

The sharp increase in current and efficiency (Fig. 4a,b) beyond 80 nm shows the unusual and unexpected behavior of the solar cell even after the repeated simulation. As per convention, we cannot consider the thickness of the window layer more relative to the active or absorber layer. Simultaneously, there was a sharp decrease in fill factor due to which we did not go beyond 80 nm. The sharp increase might be due to the limitation of the PC1D simulator, or the junction can have a breakdown^56^ thereby breaking all the bonds thereby producing a large number of electron–hole pairs. However, this behavior is well stated by Sun et al. 2012 who have fabricated a ZnSe layer of nearly 40 nm thickness. They have found that the ZnSe single layers show eminently larger photocurrent density, remarkably higher incident photon-to-current efficiency (IPCE) of about 42.5% (bulk counterpart has 0.25%) with much better photo-stability due to the combined effect of morphology and electronics on a macro-to-micro scale^54^. They have shown unique and unusual electronic structures for ultrathin thickness along with their higher carrier mobility (t = d2/k2D (d is the particle size, k is a constant, D is the diffusion coefficient of electron–hole pairs)^57^ and well-connected grain boundary^58,59^. The charge transfer resistance of the four atomic layers was lowest resulting in much higher carrier transport with a low corrosion rate^54^. Their synergistic surface distortion leads them to photostability. The contact with ZnO, ITO (Indium tin Oxide), etc. eases the electron to flow in the external circuit. This is not possible with the bulk counterpart of the ZnSe or in the presence of molecules. They have examined the distorted surface with X-ray absorption fine structure (XAFS) showing their unique and excellent structural stability, enhanced photoconversion efficiency, and photostability. 2.14 mAcm^−2^ of photocurrent density was achieved which is 195 times higher than that of its bulk form. They have reported their result mentioning that this behavior of the ZnSe thin layer has opened new avenues for bringing on a series of unprecedented excellent properties^54^.

In their experiment, the ZnSe was in contact with ITO. Stolarska et al. in 2017 found that ZnO is a robust alternative material for ITO replacement regarding environmental load and energy efficiency of the deposition process through the life cycle assessment technique. It is also crucial for sustainable transparent conductive oxide layer production. It is called a life cycle assessment (LCA) technique^60^. The result obtained in our simulation work almost agrees well with the above literature.

### Impact of the doping concentration of absorber and window layers

**Figure 5:**
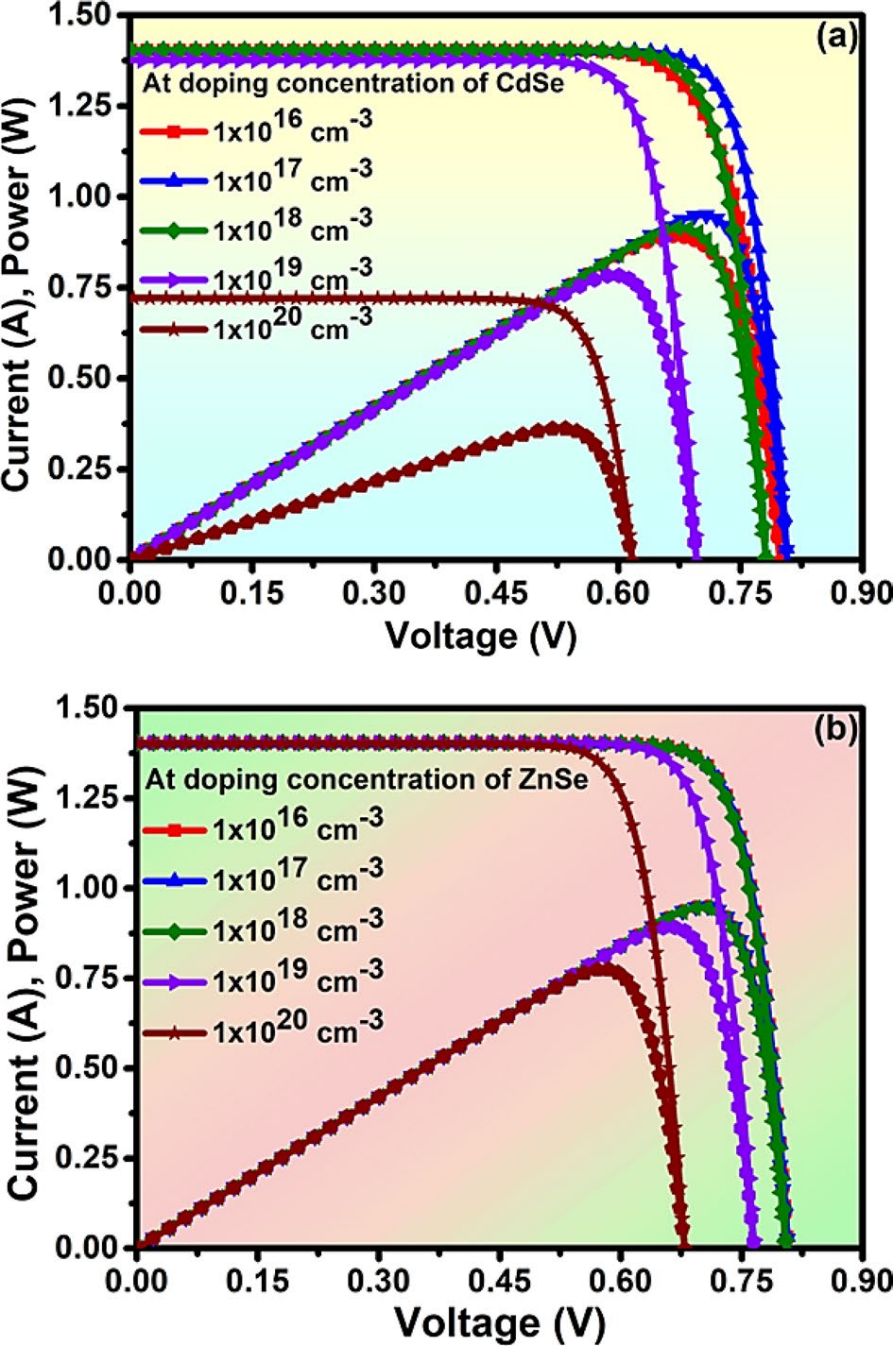
Analysis of the doping concentration of (**a**) Absorber layer (CdSe p-type), (**b**) window layer (ZnSe n-type) of solar cell.

The performance of the solar cell depends on the doping concentration on the different layers of the solar cell^61^. In this work, the CdSe absorber layer is of p-type doping and the ZnSe window layer is of n-type doping. The impact of the doping concentration of the absorber and window layer on electrical properties like current & power has been analyzed by varying 1 × 10^16^–1 × 10^20^ cm^−3^. The highest value of current (I) = 1.402 A and power (P) = 0.952 W has been obtained at optimized doping concentration 1 × 10^17^ cm^−3^ in the absorber layer as in Fig. 5a. Similarly, the highest value of current (I) = 1.404 A and power (P) = 0.901 W has been obtained at optimized doping concentration 1 × 10^17^ cm^−3^ in the window layer as in Fig. 5b. The optimized value of doping concentration 1 × 10^17^ cm^−3^ was well satisfied in both cases of the absorber and window layer.

The drastic reduction in performance caused by higher doping concentration in degenerate semiconductors is mainly due to the increased carrier scattering, Auger recombination, Fermi level pinning, non-ideal band structure, etc^62–64^. The optimization of the doping concentration is necessary for a balanced increasing carrier concentration and minimizing their harmful effect on the device performance. Further, optimization set compatibility between the input and output parameters for obtaining the overall performance of the system. So, greater care is taken at the time of optimization with respect to its objective, simulation modeling, performance metrics, and analyzing the results. The data are always reiterated, refined, and validated. The thickness of the absorber and window layer is focused for better performance by the whole system.

The optimization of the window layer thickness depends on antireflection properties which are 50 nm (i.e. 50 × 10^–9^ m) as the optimized thickness between 30 to 100 nm. The absorber of CdSe varied between 0.5 to 3 μm (i.e. 50 × 10^–6^ m) and is optimized at 2 μm. Thickness is also related to the refractive index or optical energy density of the material. The window layer should ensure the absorption of the light and transmit it to the absorber layer. The chosen thickness facilitates the efficient extraction and collection of charge carriers from the absorber layer. At this thickness, the recombination rate is minimum and provides an effective path for the charge carriers to the electrode. This layer should have adequate electrical conductivity for efficient charge transport or low sheet resistance along with material compatibility. This thickness is feasible for the deposition of nearly 5 atomic layers of ZnSe over the CdSe substrate which we have practiced recently for the thinner MXene Oxide by Pulse Laser Deposition Method and analyzed the surface with RHEED Technique^65^.

### Impact of the thickness of the ARC layer

**Figure 6:**
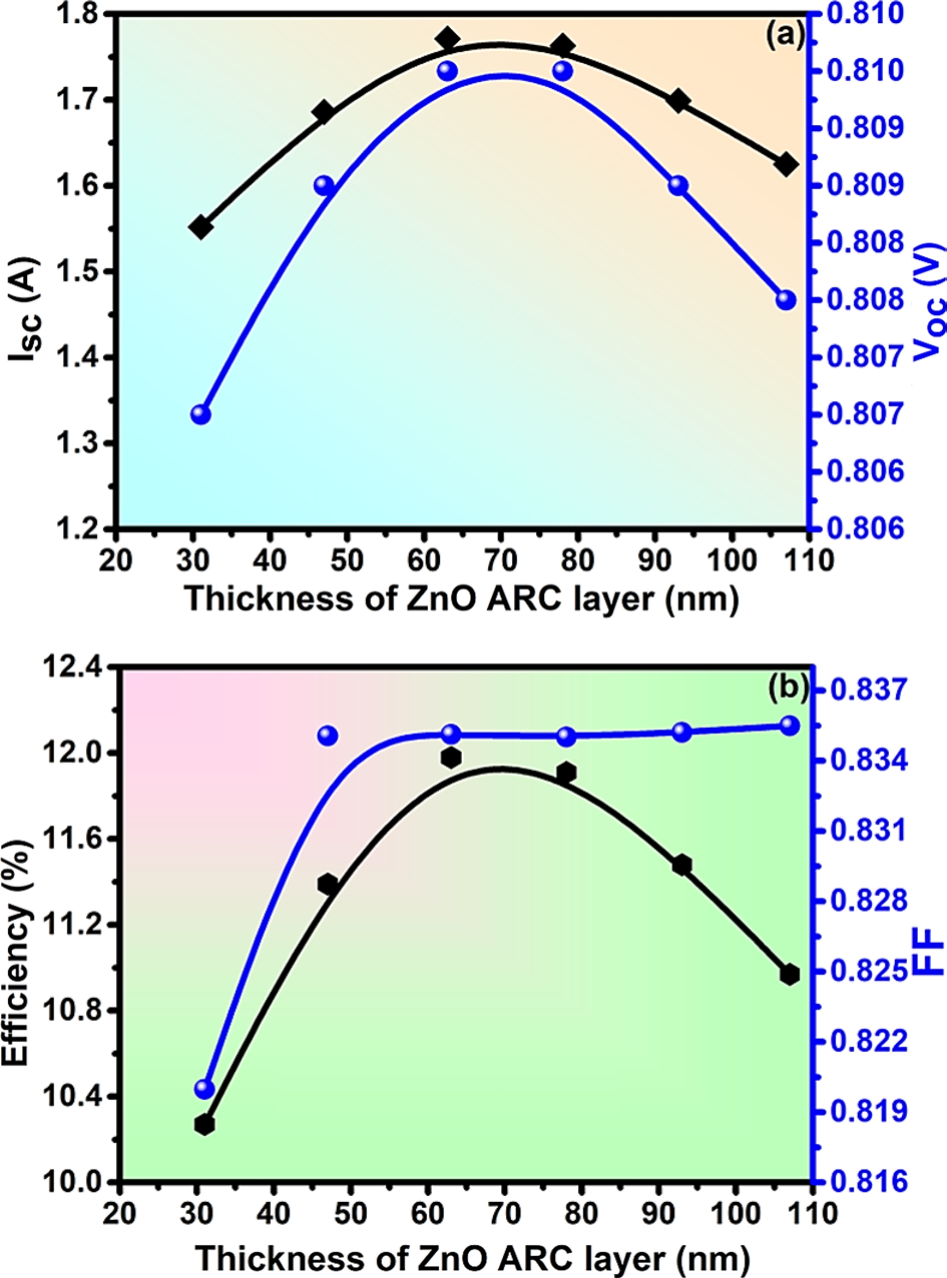
Analysis of (**a**) Isc & Voc (**b**) Efficiency & Fill factor with the thickness of the ZnO ARC layer of solar cell.

The efficiency of solar cells can be enhanced by adding an ARC layer which decreases the reflectance of solar radiation^66,67^. The impact of the thickness of the ARC layer on PV properties such as I_sc_, V_oc_, efficiency, and FF has been evaluated by varying the thickness from 31 to 107 nm and optimized at 78 nm. The numerical values of I_sc_ = 1.76 A, V_oc_ = 81 V, PCE = 11.92%, and FF = 83.5% have been achieved at the optimized thickness of the ARC as in Fig. 6.

The non-uniform thickness, defects, contaminations in the deposition chamber, traditional cleaning process, and poor film of ZnO affect the ARC by scattering the light^68^. For its improvement, the substrate should be compatible with respect to its thermal expansion coefficient, crystal structure, and the resulting strain and defects in the film. The nucleation and growth control are other measures for a good film^65^. A suitable deposition technique should be adopted under controlled temperature, pressure, and flow rate of the depositing material along with the rate of deposition. Consequently, the post-deposition annealing for quality crystal formation should be optimized. The thickness of the deposition should be uniform. The deposition technique that we recently used for the formation of an oxide layer in the preparation of MXene Oxide is preferable to meet this challenge^65^. The preparation of the single-layered ARC (SLARC) ZnO deposition on the ZnSe–CdSe solar cell can be achieved through a novel technique so far adopted in this field. The enhanced light trapping, wide bandgap engineering, simplified device structure, compatibility with ZnO as transparent conductive oxide, and scalability-versatility can lead to the novelty of this type of heterojunction solar cell.

The effect of increasing ARC thickness and electrical output depends on the device model and its material. The optimization of the ARC thickness can enhance the light absorption by optical interference, impedance matching, and reducing reflection loss^3^. However, care should be taken on the increased series resistance that increases the voltage loss, thereby reducing the fill factor and efficiency as a whole^3^. Consequently, the collection of charge carriers produced in the active layer will be reduced with the increase of the recombination rates or the longer carrier diffusion length. Similarly, there is the probability of blocking some portion of incident light from reaching the active layer^3^. With the advancement of technologies, the deposition of such a thinner layer of 50 nm (~ 5 atomic thickness)^69^ is feasible for experiments as well which we have incorporated for a very sensitive MXene layer recently and analyzed the surface^65^.

### Analysis of optoelectrical properties

**Figure 7:**
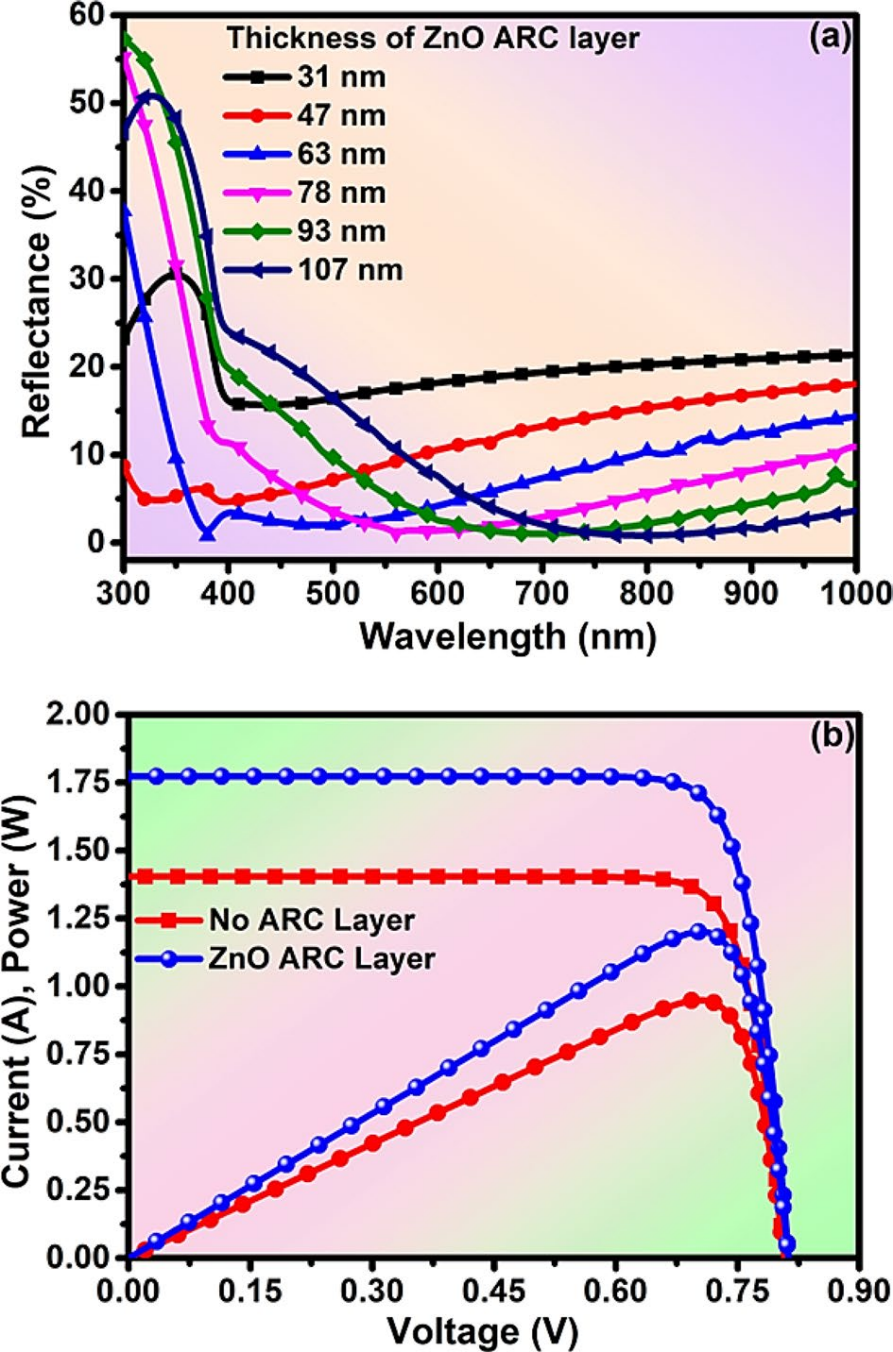
Analysis of (**a**) reflectance with the thickness of ARC layer (**b**) I, P–V characteristics of the with or without ARC layer of solar cell.

**Figure 8:**
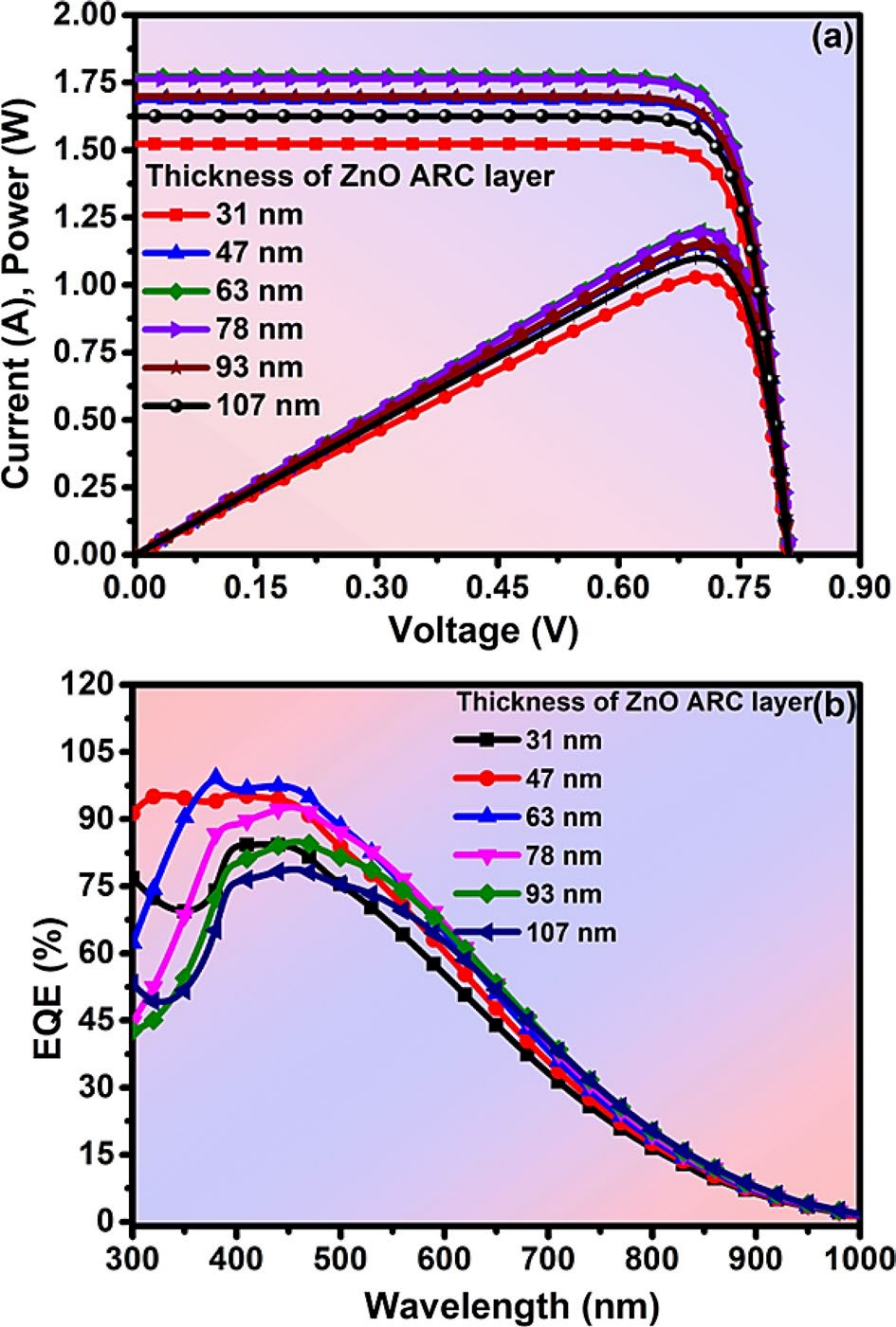
Analysis of (**a**) I, P–V curve (**b**) EQE at the different thicknesses of the ARC layer of solar cell.

The reflectance of solar radiation on the surface of solar cells plays a vital role to enhance the rate of generation of photocurrent. The ARC layer on the surface of the solar cell helps to absorb incident photons, reduces reflectance, and increases the I_sc_ due to destructive interference^67,70^**.** The appropriate thickness of the ARC layer can only produce destructive interference. The thickness (d_1_) of the ARC layer to get a quarter-wavelength coating of a transparent material is given by^71^,

1 $${\text{d}}_{1} = \frac{{{\uplambda }_{0} }}{{4{\upeta }_{1} }}$$

and,

2 $${\upeta }_{1} = \sqrt {{\upeta }_{0} {\upeta }_{2} }$$

where η_1,_ ${\upeta }_{0, } and {\upeta }_{2}$ are the refractive indices of the coating material, air, and window layer respectively, and λ_0_ is the wavelength of the incident light at a wavelength. By using Eqs. (1) and (2), we have calculated the thickness of the ZnO ARC layer. The reflectance spectra of different thicknesses of the ZnO ARC layer have been explored between 300 and 1000 nm wavelength. The average reflectance of 18.91%, 12.2%, 7.53%, 6.45%, 6.61%, and 8.07% at a thickness of 31 nm, 47 nm, 63 nm, 78 nm, 93 nm, and 107 nm respectively in the range of 400–1000 nm wavelength as in Fig. 7a. The minimum average minimum reflectance (R_av_) of 6.45% has been obtained at 78 nm thickness of the ZnO ARC layer due to perfect destructive interference^72^**.** The I–V and P–V characteristics of solar cells with and without ARC layers have been analyzed with an optimized thickness of the ARC layer respectively as in Fig. 7b. The numerical values of I = 1.77 A & P = 1.2 W have been exhibited with the ARC layer, whilst, I = 1.4 A & P = 0.95 W without the ARC layer as in Fig. 7b, which indicates that deposition of ARC layer has enhanced the PCE of the solar cell. Additionally, the I–V and P–V characteristics of solar cells have been analyzed at the different thicknesses of the ARC layer as in Fig. 7a. The numerical values of I = 1.77 A & P = 1.2 W have been exhibited at the optimized thickness of the ARC layer as in Fig. 8a. The reported PV properties of ZnSe-CdSe solar cells have been summarized in Table 2 and the optimized and recommended are in Table 3. Furthermore, the more detailed behavior of the solar cell has been analyzed by the external quantum efficiency (EQE) method in a specified range of wavelength (300–1000 nm)^73,74^. The EQE of solar cells has been observed at about 90% at a particular wavelength at 470 nm (visible light range) as in Fig. 8b. Therefore, this simulation study of optoelectrical properties manifests that the ZnSe–CdSe Solar cell-based with ZnO ARC layer is cost-effective attaining an efficiency of nearly 12% and stimulated with visible light.

**Table 2:** Reported PV properties of ZnSe-CdSe solar cell.

Structure of Solar cell	Isc (mA/cm^2^)	Voc (V)	FF (%)	Efficiency (%)	References
SnO2/CdSe/CdTe/Al	4.32	0.87	76.19	16.13	^76^
CdSe/CdTe	19.89	0.96	78.22	15	^77^
CdS/CdSe/ZnS	4.79	0.76	0.42	1.5	^78^
ITO/ZnS/CdSe	13.82	1.38	90.08	17.35	^20^
ZnO/ZnSe/CdSe	17.70	0.81	90.08	11.98	This work

**Table 3:** Summary of optimized parameters of ZnSe-CdSe Solar cell.

Cell parameters	Optimized values	
	Absorber layer (CdSe)	Window layer (ZnSe)
Thickness	2 μm	50 nm
Doping Concentration	1 × 10^17^ cm^−3^	1 × 10^17^ cm^−3^
Thickness of ARC layer (ZnO)	78 nm	

## Conclusion

The numerical analysis of optoelectrical properties of ZnSe–CdSe solar cells has been successfully investigated by using the PC1D simulation tool. We have studied the band alignment and band offset across the heterojunction of the solar cell. The electron affinity of ZnSe (~ − 0.64 eV) is lower than that of CdSe (~ − 0.50 eV) indicating the possibility of positive CBO with ΔEc = 0.25 eV and negative VBO with ΔEv = 1.2–2.16 = − 0.96 eV. The positive CBO prevents the flow of electrons from the CdSe to the ZnSe layer. CBOF of The simulation results reveals the increasing efficiency of solar cells by adding an antireflection coating. The photovoltaic properties have been optimized by varying thicknesses of the p-CdSe absorber layer, n-ZnSe window layer, and ZnO ARC layer and also investigated the doping concentration effect on solar cell performance. The minimum average minimum reflectance (R_av_) of 6.45% has been obtained at 63 nm thickness of the ZnO ARC layer due to perfect destructive interference. The ZnO/ZnSe/CdSe solar cell exhibited a high efficiency of 11.98% with I_sc_ = 1.72 A, V_oc_ = 0.81 V, and FF = 90.8% at an optimized thickness of 2 μm absorber layer, 50 nm window layer, and 78 nm ARC layer. The efficiency and short circuit current increase rapidly and unusually after 80 nm thickness of the ZnSe window layer indicating the possibility of the production of a large number of electron–hole pairs due to the combined effect of morphology and electronics on a macro-to-micro scale. which is in good agreement with the previous literature. Thus, the optoelectrical properties from this study exhibited that the ZnSe–CdSe Solar cell-based with ZnO ARC layer is cheap, visible light stimulated, and efficient to fabricate high-performance solar cells within the optimized limit.

## Supplementary Information

Supplementary Table 1.

Supplementary Table 2.

## Data Availability

The datasets used and/or analyzed during the current study are available from the corresponding author upon reasonable request.
