# Supplementary material for: Numerical assessment of optoelectrical properties of ZnSe–CdSe solar cell-based with ZnO antireflection coating layer
Source: Sci Rep. 2023 Jul 27;13:12193. doi: 10.1038/s41598-023-38906-z (PMC10374893; doi:10.1038/s41598-023-38906-z)
Supplement: Supplementary file 1 — Supplementary Table 1. [file 41598_2023_38906_MOESM1_ESM.docx]

**Some important tables of the simulated data**

| **p-CdSe** | | | | **n-ZnSe** | | | |
| --- | --- | --- | --- | --- | --- | --- | --- |
| **Thickness (μm)** | **Isc** | **Voc** | **Eff** | **Thickness (nm)** | **Isc** | **Voc** | **Eff** |
| 0.6 | 0.7919 | 0.8136 | 6.040 | 10 | 1.392 | 0.8075 | 9.430 |
| 0.8 | 0.9181 | 0.8124 | 6.390 | 20 | 1.395 | 0.8069 | 9.410 |
| 1.0 | 1.026 | 0.8111 | 7.030 | 30 | 1.398 | 0.8063 | 9.452 |
| 1.200 | 1.120 | 0.8099 | 7.650 | 40 | 1.401 | 0.8058 | 9.462 |
| 1.400 | 1.203 | 0.8086 | 8.190 | 50 | 1.404 | 0.8052 | 9.473 |
| 1.600 | 1.277 | 0.8074 | 8.660 | 60 | 1.407 | 0.8047 | 9.483 |
| 1.800 | 1.344 | 0.8063 | 9.090 | 70 | 1.410 | 0.8042 | 9.490 |
| 2.0 | 1.404 | 0.8052 | 9.470 | 80 | 1.412 | 0.8037 | 9.500 |
| 2.200 | 1.459 | 0.8042 | 9.810 | 90 | 1.415 | 0.8032 | 9.510 |
| 2.400 | 1.509 | 0.8032 | 10.130 | 100 | 1.628 | 0.7786 | 10.510 |
| 2.600 | 1.555 | 0.8022 | 10.410 |  |  |  |  |
| 2.800 | 1.598 | 0.8013 | 10.680 |  |  |  |  |
| 3.0 | 1.638 | 0.8004 | 10.920 |  |  |  |  |

| **Quantum Efficiency** | | | | | | |
| --- | --- | --- | --- | --- | --- | --- |
| Thickness | 31nm | 47 | 63 | 78 | 93 nm | 107nm |
| Primary Source Wavelength | Pri-Surface Total Reflectance | Pri-Surface Total Reflectance | Pri-Surface Total Reflectance | Pri-Surface Total Reflectance | Pri-Surface Total Reflectance | Pri-Surface Total Reflectance |
| 300 | 23.1697 | 8.72989 | 37.7323 | 55.2242 | 57.2998 | 46.6576 |
| 300 | 23.1697 | 8.72989 | 37.7323 | 55.2242 | 57.2998 | 46.6576 |
| 310 | 25.5981 | 6.17446 | 31.5511 | 51.5212 | 56.3743 | 49.1763 |
| 320 | 27.7104 | 4.96891 | 25.6645 | 47.4922 | 54.9246 | 50.6723 |
| 330 | 29.2753 | 4.830 | 19.8753 | 42.7847 | 52.6421 | 50.9694 |
| 340 | 30.2606 | 4.80077 | 14.435 | 37.4425 | 49.5177 | 50.1655 |
| 350 | 30.6137 | 5.30528 | 9.58705 | 31.5588 | 45.5136 | 48.2792 |
| 360 | 30.2324 | 5.85129 | 5.660 | 25.3102 | 40.5788 | 45.2444 |
| 370 | 28.8995 | 6.18376 | 2.50942 | 19.0179 | 34.677 | 40.8888 |
| 380 | 26.0511 | 6.005 | 0.776866 | 13.3252 | 27.8632 | 34.8439 |
| 390 | 17.6699 | 4.56545 | 2.80366 | 11.354 | 20.8561 | 25.2772 |
| 400 | 15.8555 | 4.62933 | 3.55367 | 11.450 | 19.8493 | 23.9332 |
| 410 | 15.7679 | 4.85104 | 3.24388 | 10.890 | 18.8426 | 23.4429 |
| 420 | 15.7016 | 5.05704 | 2.96576 | 9.42688 | 17.8482 | 22.9007 |
| 430 | 15.6642 | 5.25864 | 2.71707 | 8.55684 | 16.855 | 22.3016 |
| 440 | 15.6608 | 5.46565 | 2.49892 | 7.72248 | 15.8555 | 21.6418 |
| 450 | 15.6948 | 5.68622 | 2.31438 | 6.92176 | 14.8463 | 20.9191 |
| 460 | 15.768 | 5.92687 | 2.16745 | 6.15574 | 13.8269 | 20.1332 |
| 470 | 15.8811 | 6.19261 | 2.06235 | 5.42763 | 12.950 | 19.286 |
| 480 | 16.0337 | 6.48707 | 2.00309 | 4.742 | 11.850 | 18.3809 |
| 490 | 16.2227 | 6.81122 | 1.99314 | 4.10555 | 10.160 | 17.4244 |
| 500 | 16.4274 | 7.15367 | 2.03373 | 3.53346 | 9.74053 | 16.4354 |
| 510 | 16.6316 | 7.50325 | 2.12117 | 3.03422 | 8.78289 | 15.4325 |
| 520 | 16.8295 | 7.85411 | 2.980 | 2.60817 | 7.87786 | 14.4292 |
| 530 | 17.0201 | 8.20377 | 2.41409 | 2.25206 | 7.02957 | 13.4349 |
| 540 | 17.2036 | 8.55077 | 2.60904 | 1.96173 | 6.24027 | 12.720 |
| 550 | 17.3802 | 8.89397 | 2.83014 | 1.73282 | 5.51125 | 11.280 |
| 560 | 17.5504 | 9.23241 | 3.07332 | 1.090 | 4.843 | 10.730 |
| 570 | 17.7142 | 9.56534 | 3.33494 | 1.44154 | 4.23528 | 9.68539 |
| 580 | 17.8721 | 9.89216 | 3.61175 | 1.37037 | 3.68726 | 8.83113 |
| 590 | 18.0241 | 10.240 | 3.90087 | 1.34318 | 3.19758 | 8.01764 |
| 600 | 18.1707 | 10.580 | 4.19974 | 1.35588 | 2.450 | 7.740 |
| 610 | 18.3119 | 10.832 | 4.50611 | 1.460 | 2.38592 | 6.52221 |
| 620 | 18.448 | 11.1309 | 4.81802 | 1.48569 | 2.05951 | 5.84329 |
| 630 | 18.5793 | 11.250 | 5.13374 | 1.570 | 1.78273 | 5.21134 |
| 640 | 18.706 | 11.650 | 5.45177 | 1.73144 | 1.55293 | 4.62658 |
| 650 | 18.8282 | 11.320 | 5.77079 | 1.88993 | 1.36736 | 4.08881 |
| 660 | 18.9461 | 12.240 | 6.0897 | 2.06841 | 1.22328 | 3.59749 |
| 670 | 18.9462 | 12.430 | 6.40751 | 2.26437 | 1.11791 | 3.15175 |
| 680 | 19.17 | 12.769 | 6.72341 | 2.47549 | 1.04853 | 2.75047 |
| 690 | 19.2762 | 13.0166 | 7.03668 | 2.69964 | 1.01247 | 2.39232 |
| 700 | 19.3789 | 13.2573 | 7.34674 | 2.93492 | 1.00715 | 2.07578 |
| 710 | 19.4782 | 13.4911 | 7.65307 | 3.17957 | 1.03007 | 1.79922 |
| 720 | 19.5742 | 13.7183 | 7.95526 | 3.43202 | 1.07886 | 1.56088 |
| 730 | 19.667 | 13.939 | 8.25297 | 3.69083 | 1.15124 | 1.35895 |
| 740 | 19.7569 | 14.1533 | 8.54593 | 3.95474 | 1.24507 | 1.19156 |
| 750 | 19.8439 | 14.3616 | 8.390 | 4.22258 | 1.35831 | 1.05684 |
| 760 | 19.9281 | 14.5638 | 9.11673 | 4.49333 | 1.48905 | 0.952879 |
| 770 | 20.0097 | 14.7603 | 9.39427 | 4.76608 | 1.63553 | 0.877823 |
| 780 | 20.0887 | 14.9511 | 9.66645 | 5.03999 | 1.79606 | 0.829825 |
| 790 | 20.1653 | 15.1365 | 9.93318 | 5.31436 | 1.910 | 0.807085 |
| 800 | 20.2396 | 15.3166 | 10.450 | 5.58852 | 2.15322 | 0.807853 |
| 810 | 20.3116 | 15.4915 | 10.030 | 5.86192 | 2.34708 | 0.830437 |
| 820 | 20.3815 | 15.6615 | 10.060 | 6.13406 | 2.54945 | 0.87321 |
| 830 | 20.4493 | 15.8268 | 10.550 | 6.650 | 2.75921 | 0.934613 |
| 840 | 20.5152 | 15.9873 | 11.185 | 6.67286 | 2.97531 | 1.01316 |
| 850 | 20.5791 | 16.1434 | 11.920 | 6.93881 | 3.680 | 1.10743 |
| 860 | 20.6411 | 16.2952 | 11.810 | 7.20207 | 3.280 | 1.21608 |
| 870 | 20.7014 | 16.4427 | 11.180 | 7.46238 | 3.65253 | 1.33785 |
| 880 | 20.7601 | 16.5862 | 12.0904 | 7.71955 | 3.88524 | 1.47154 |
| 890 | 20.817 | 16.7257 | 12.304 | 7.97339 | 4.12028 | 1.61602 |
| 900 | 20.8724 | 16.8615 | 12.270 | 8.22376 | 4.35704 | 1.77023 |
| 910 | 20.9263 | 16.9936 | 12.650 | 8.47053 | 4.59499 | 1.320 |
| 920 | 20.9787 | 17.1221 | 12.560 | 8.71362 | 4.83362 | 2.10398 |
| 930 | 21.0297 | 17.2472 | 13.110 | 8.95295 | 5.0725 | 2.28173 |
| 940 | 21.0794 | 17.369 | 13.300 | 9.18845 | 5.31121 | 2.46564 |
| 950 | 21.1277 | 17.4875 | 13.4855 | 9.42009 | 5.54941 | 2.65496 |
| 960 | 21.1748 | 17.603 | 13.6667 | 9.64783 | 5.78676 | 2.849 |
| 970 | 21.2206 | 17.7154 | 13.8436 | 9.87167 | 6.02297 | 3.04712 |
| 980 | 21.2653 | 17.825 | 14.0165 | 10.0916 | 7.780 | 3.24872 |
| 990 | 21.3089 | 17.9317 | 14.1853 | 10.760 | 6.491 | 3.45326 |
| 1000 | 21.3513 | 18.0357 | 14.3502 | 10.980 | 6.72238 | 3.66023 |
| 1010 | 21.3927 | 18.1371 | 14.5113 | 10.728 | 6.95176 | 3.86917 |
| 1020 | 21.4331 | 18.2359 | 14.6687 | 10.250 | 7.17898 | 4.07964 |
| 1030 | 21.4724 | 18.3323 | 14.8224 | 11.1331 | 7.390 | 4.29125 |
| 1040 | 21.5108 | 18.4262 | 14.9727 | 11.330 | 7.62641 | 4.50365 |
| 1050 | 21.5483 | 18.5179 | 15.1195 | 11.320 | 7.640 | 4.71649 |
| 1060 | 21.5849 | 18.6073 | 15.2629 | 11.270 | 8.06379 | 4.92949 |
| 1070 | 21.6207 | 18.6946 | 15.4031 | 11.860 | 8.27849 | 5.14236 |
| 1080 | 21.6555 | 18.7797 | 15.5401 | 12.0808 | 8.49042 | 5.35483 |
| 1090 | 21.6896 | 18.8627 | 15.674 | 12.960 | 8.69954 | 5.56667 |
| 1100 | 21.7228 | 18.9438 | 15.8048 | 12.490 | 8.90581 | 5.77767 |
| 1110 | 21.7552 | 19.0229 | 15.9327 | 12.670 | 9.10918 | 5.98765 |
| 1120 | 21.7868 | 19.1001 | 16.0577 | 12.560 | 9.30961 | 6.19641 |
| 1130 | 21.8177 | 19.1754 | 16.1799 | 12.030 | 9.50708 | 6.40378 |
| 1140 | 21.8478 | 19.2489 | 16.2993 | 13.1022 | 9.70157 | 6.60964 |
| 1150 | 21.8772 | 19.3207 | 16.416 | 13.2608 | 9.89308 | 6.81384 |
| 1160 | 21.9059 | 19.3908 | 16.5301 | 13.4162 | 10.0816 | 7.01629 |
| 1170 | 21.9339 | 19.4592 | 16.6416 | 13.5686 | 10.720 | 7.21687 |
| 1180 | 21.9612 | 19.526 | 16.7507 | 13.7179 | 10.980 | 7.550 |
| 1190 | 21.9878 | 19.5913 | 16.8573 | 13.8642 | 10.940 | 7.61211 |
| 1200 | 22.0138 | 19.655 | 16.9616 | 14.0076 | 10.610 | 7.80663 |

| **No ARC** | | | **with ZnO ARC** | | |
| --- | --- | --- | --- | --- | --- |
| Base Current & Power | | | Base Current & Power | | |
| Base Voltage | Base Current | Base Power | Base Voltage | Base Current | Base Power |
| -0.719227 | -1.40386 | 1.0097 | -0.645424 | -1.77288 | 1.14426 |
| -0.719227 | -1.40386 | 1.0097 | -0.645424 | -1.77288 | 1.14426 |
| -0.699228 | -1.40386 | 0.981619 | -0.625424 | -1.77288 | 1.1088 |
| -0.679228 | -1.40386 | 0.953542 | -0.605424 | -1.77288 | 1.07334 |
| -0.659228 | -1.40386 | 0.925465 | -0.585424 | -1.77288 | 1.03789 |
| -0.639228 | -1.40386 | 0.897387 | -0.565424 | -1.77288 | 1.00243 |
| -0.619228 | -1.40386 | 0.86931 | -0.545424 | -1.77288 | 0.966971 |
| -0.599228 | -1.40386 | 0.841233 | -0.525424 | -1.77288 | 0.931514 |
| -0.579228 | -1.40386 | 0.813156 | -0.505424 | -1.77288 | 0.896056 |
| -0.559228 | -1.40386 | 0.785078 | -0.485424 | -1.77288 | 0.860598 |
| -0.539228 | -1.40386 | 0.757001 | -0.465424 | -1.77288 | 0.825141 |
| -0.519228 | -1.40386 | 0.728924 | -0.445424 | -1.77288 | 0.789683 |
| -0.499228 | -1.40386 | 0.700847 | -0.425424 | -1.77288 | 0.754226 |
| -0.479228 | -1.40386 | 0.672769 | -0.405424 | -1.77288 | 0.718768 |
| -0.459228 | -1.40386 | 0.644692 | -0.385424 | -1.77288 | 0.68331 |
| -0.439228 | -1.40386 | 0.616615 | -0.365424 | -1.77288 | 0.647853 |
| -0.419228 | -1.40386 | 0.588538 | -0.345424 | -1.77288 | 0.612395 |
| -0.399228 | -1.40386 | 0.56046 | -0.325424 | -1.77288 | 0.576938 |
| -0.379228 | -1.40386 | 0.532383 | -0.305424 | -1.77288 | 0.54148 |
| -0.359228 | -1.40386 | 0.504306 | -0.285424 | -1.77288 | 0.506023 |
| -0.339228 | -1.40386 | 0.476229 | -0.265424 | -1.77288 | 0.470565 |
| -0.319228 | -1.40386 | 0.448151 | -0.245424 | -1.77288 | 0.435107 |
| -0.299228 | -1.40386 | 0.420074 | -0.225424 | -1.77288 | 0.39965 |
| -0.279228 | -1.40386 | 0.391997 | -0.205424 | -1.77288 | 0.364192 |
| -0.259228 | -1.40386 | 0.36392 | -0.185424 | -1.77288 | 0.328735 |
| -0.239228 | -1.40386 | 0.335843 | -0.165424 | -1.77288 | 0.293277 |
| -0.219228 | -1.40386 | 0.307765 | -0.145424 | -1.77288 | 0.257819 |
| -0.199228 | -1.40386 | 0.279688 | -0.125424 | -1.77288 | 0.222362 |
| -0.179228 | -1.40386 | 0.251611 | -0.105424 | -1.77288 | 0.186904 |
| -0.159228 | -1.40386 | 0.223534 | -0.0854241 | -1.77288 | 0.151447 |
| -0.139228 | -1.40386 | 0.195456 | -0.0654241 | -1.77288 | 0.115989 |
| -0.119228 | -1.40386 | 0.167379 | -0.0454241 | -1.77288 | 0.0805315 |
| -0.0992277 | -1.40386 | 0.139302 | -0.0254242 | -1.77288 | 0.0450739 |
| -0.0792277 | -1.40386 | 0.111225 | -0.00542416 | -1.77288 | 0.00961638 |
| -0.0592277 | -1.40386 | 0.0831475 | 0.0145758 | -1.77288 | -0.0258412 |
| -0.0392277 | -1.40386 | 0.0550702 | 0.0345758 | -1.77288 | -0.0612988 |
| -0.0192277 | -1.40386 | 0.026993 | 0.0545758 | -1.77288 | -0.0967563 |
| 0.00077231 | -1.40386 | -0.00108422 | 0.0745758 | -1.77288 | -0.132214 |
| 0.0207723 | -1.40386 | -0.0291614 | 0.0945758 | -1.77288 | -0.167671 |
| 0.0407723 | -1.40386 | -0.0572387 | 0.114576 | -1.77288 | -0.203129 |
| 0.0607723 | -1.40386 | -0.0853159 | 0.134576 | -1.77288 | -0.238587 |
| 0.0807723 | -1.40386 | -0.113393 | 0.154576 | -1.77288 | -0.274044 |
| 0.100772 | -1.40386 | -0.14147 | 0.174576 | -1.77288 | -0.309502 |
| 0.120772 | -1.40386 | -0.169548 | 0.194576 | -1.77288 | -0.344959 |
| 0.140772 | -1.40386 | -0.197625 | 0.214576 | -1.77288 | -0.380417 |
| 0.160772 | -1.40386 | -0.225702 | 0.234576 | -1.77288 | -0.415874 |
| 0.180772 | -1.40386 | -0.253779 | 0.254576 | -1.77288 | -0.451332 |
| 0.200772 | -1.40386 | -0.281856 | 0.274576 | -1.77288 | -0.486789 |
| 0.220772 | -1.40386 | -0.309933 | 0.294575 | -1.77288 | -0.522246 |
| 0.240772 | -1.40386 | -0.338011 | 0.314575 | -1.77288 | -0.557703 |
| 0.260772 | -1.40386 | -0.366088 | 0.334575 | -1.77288 | -0.59316 |
| 0.280772 | -1.40386 | -0.394165 | 0.354575 | -1.77287 | -0.628617 |
| 0.300772 | -1.40386 | -0.422242 | 0.374575 | -1.77287 | -0.664073 |
| 0.320772 | -1.40386 | -0.450319 | 0.394574 | -1.77287 | -0.699528 |
| 0.340772 | -1.40386 | -0.478395 | 0.414573 | -1.77286 | -0.734982 |
| 0.360771 | -1.40386 | -0.506471 | 0.434571 | -1.77286 | -0.770433 |
| 0.380771 | -1.40385 | -0.534547 | 0.454569 | -1.77285 | -0.805881 |
| 0.40077 | -1.40385 | -0.562622 | 0.474565 | -1.77283 | -0.841322 |
| 0.420769 | -1.40385 | -0.590696 | 0.494559 | -1.77279 | -0.876751 |
| 0.440768 | -1.40384 | -0.618767 | 0.514548 | -1.77274 | -0.912159 |
| 0.460766 | -1.40383 | -0.646835 | 0.534528 | -1.77264 | -0.947526 |
| 0.480762 | -1.40381 | -0.674897 | 0.554492 | -1.77246 | -0.982815 |
| 0.500755 | -1.40378 | -0.702948 | 0.574424 | -1.77212 | -1.01795 |
| 0.520744 | -1.40372 | -0.730978 | 0.594292 | -1.77146 | -1.05276 |
| 0.540724 | -1.40362 | -0.75897 | 0.614032 | -1.77016 | -1.08693 |
| 0.560687 | -1.40344 | -0.786888 | 0.633516 | -1.76758 | -1.11979 |
| 0.580617 | -1.40309 | -0.814657 | 0.652508 | -1.76254 | -1.15007 |
| 0.600482 | -1.40241 | -0.842124 | 0.670617 | -1.75308 | -1.17565 |
| 0.620216 | -1.40108 | -0.868973 | 0.687326 | -1.73663 | -1.19363 |
| 0.639689 | -1.39844 | -0.894569 | 0.702169 | -1.71085 | -1.2013 |
| 0.658659 | -1.39329 | -0.917705 | 0.714952 | -1.67476 | -1.19737 |
| 0.67673 | -1.38365 | -0.936357 | 0.725797 | -1.62898 | -1.18231 |
| 0.693384 | -1.36692 | -0.947801 | 0.735005 | -1.57503 | -1.15765 |
| 0.70816 | -1.3408 | -0.949503 | 0.742906 | -1.51453 | -1.12515 |
| 0.720875 | -1.30438 | -0.940293 | 0.74978 | -1.4489 | -1.08636 |
| 0.731661 | -1.25831 | -0.920653 | 0.75585 | -1.37925 | -1.04251 |
| 0.740821 | -1.20411 | -0.892029 | 0.761284 | -1.30642 | -0.994558 |
| 0.748684 | -1.14342 | -0.856059 | 0.766209 | -1.23104 | -0.943236 |
| 0.755529 | -1.07764 | -0.81419 | 0.770719 | -1.1536 | -0.8891 |
| 0.761575 | -1.00788 | -0.767574 | 0.774891 | -1.07445 | -0.832584 |
| 0.766991 | -0.934953 | -0.7171 | 0.778779 | -0.993896 | -0.774026 |
| 0.7719 | -0.859499 | -0.663447 | 0.78243 | -0.912152 | -0.713695 |
| 0.776398 | -0.781992 | -0.607137 | 0.78588 | -0.8294 | -0.651809 |
| 0.780559 | -0.702797 | -0.548574 | 0.789157 | -0.745784 | -0.588541 |
| 0.784439 | -0.622197 | -0.488076 | 0.792285 | -0.661423 | -0.524036 |
| 0.788083 | -0.540417 | -0.425894 | 0.795283 | -0.576413 | -0.458412 |
| 0.791527 | -0.457634 | -0.36223 | 0.798167 | -0.490835 | -0.391768 |
| 0.794798 | -0.373992 | -0.297249 | 0.800951 | -0.404755 | -0.324189 |
| 0.797922 | -0.289608 | -0.231085 | 0.803646 | -0.318231 | -0.255745 |
| 0.800916 | -0.204578 | -0.16385 | 0.806262 | -0.231311 | -0.186497 |
| 0.803796 | -0.118982 | -0.0956377 | 0.808807 | -0.144035 | -0.116497 |
| 0.806577 | -0.0328873 | -0.0265262 | 0.811288 | -0.0564404 | -0.0457894 |
| 0.80927 | 0.0536506 | 0.0434178 | 0.813711 | 0.0314436 | 0.025586 |
| 0.811883 | 0.140583 | 0.114137 | 0.816082 | 0.11959 | 0.0975952 |
| 0.814426 | 0.22787 | 0.185583 | 0.818405 | 0.207975 | 0.170208 |
| 0.816905 | 0.315475 | 0.257713 | 0.820684 | 0.296579 | 0.243397 |
| 0.819326 | 0.403368 | 0.33049 | 0.822924 | 0.385382 | 0.31714 |
| 0.821695 | 0.491523 | 0.403882 | 0.825126 | 0.47437 | 0.391415 |
| 0.824017 | 0.579915 | 0.47786 | 0.827294 | 0.563528 | 0.466203 |
| 0.826295 | 0.668526 | 0.552399 | 0.829431 | 0.652843 | 0.541488 |
| 0.828533 | 0.757336 | 0.627478 | 0.831539 | 0.742303 | 0.617254 |
| 0.830734 | 0.846329 | 0.703075 | 0.83362 | 0.831899 | 0.693488 |

| 78 nm | | | 93 nm | | | 107 nm | | |
| --- | --- | --- | --- | --- | --- | --- | --- | --- |
| Base Current & Power | | | Base Current & Power | | | Base Current & Power | | |
| Base Voltage | Base Current | Base Power | Base Voltage | Base Current | Base Power | Base Voltage | Base Current | Base Power |
| -0.647339 | -1.7633 | 1.14146 | -0.660176 | -1.69912 | 1.12172 | -0.67505 | -1.62475 | 1.09679 |
| -0.647339 | -1.7633 | 1.14146 | -0.660176 | -1.69912 | 1.12172 | -0.67505 | -1.62475 | 1.09679 |
| -0.627339 | -1.7633 | 1.10619 | -0.640176 | -1.69912 | 1.08774 | -0.65505 | -1.62475 | 1.06429 |
| -0.607339 | -1.7633 | 1.07092 | -0.620176 | -1.69912 | 1.05375 | -0.63505 | -1.62475 | 1.0318 |
| -0.587339 | -1.7633 | 1.03566 | -0.600176 | -1.69912 | 1.01977 | -0.61505 | -1.62475 | 0.999302 |
| -0.567339 | -1.7633 | 1.00039 | -0.580176 | -1.69912 | 0.985789 | -0.59505 | -1.62475 | 0.966807 |
| -0.547339 | -1.7633 | 0.965125 | -0.560176 | -1.69912 | 0.951807 | -0.57505 | -1.62475 | 0.934312 |
| -0.527339 | -1.7633 | 0.929859 | -0.540176 | -1.69912 | 0.917824 | -0.55505 | -1.62475 | 0.901817 |
| -0.507339 | -1.7633 | 0.894593 | -0.520176 | -1.69912 | 0.883842 | -0.53505 | -1.62475 | 0.869322 |
| -0.487339 | -1.7633 | 0.859327 | -0.500176 | -1.69912 | 0.849859 | -0.51505 | -1.62475 | 0.836827 |
| -0.467339 | -1.7633 | 0.824061 | -0.480176 | -1.69912 | 0.815877 | -0.49505 | -1.62475 | 0.804332 |
| -0.447339 | -1.7633 | 0.788795 | -0.460176 | -1.69912 | 0.781894 | -0.475051 | -1.62475 | 0.771837 |
| -0.427339 | -1.7633 | 0.753529 | -0.440176 | -1.69912 | 0.747912 | -0.455051 | -1.62475 | 0.739342 |
| -0.407339 | -1.7633 | 0.718263 | -0.420176 | -1.69912 | 0.71393 | -0.435051 | -1.62475 | 0.706847 |
| -0.387339 | -1.7633 | 0.682997 | -0.400176 | -1.69912 | 0.679947 | -0.415051 | -1.62475 | 0.674352 |
| -0.367339 | -1.7633 | 0.647731 | -0.380176 | -1.69912 | 0.645965 | -0.395051 | -1.62475 | 0.641857 |
| -0.347339 | -1.7633 | 0.612465 | -0.360176 | -1.69912 | 0.611982 | -0.375051 | -1.62475 | 0.609362 |
| -0.327339 | -1.7633 | 0.577199 | -0.340176 | -1.69912 | 0.578 | -0.355051 | -1.62475 | 0.576867 |
| -0.307339 | -1.7633 | 0.541932 | -0.320176 | -1.69912 | 0.544017 | -0.335051 | -1.62475 | 0.544372 |
| -0.287339 | -1.7633 | 0.506666 | -0.300176 | -1.69912 | 0.510035 | -0.315051 | -1.62475 | 0.511878 |
| -0.267339 | -1.7633 | 0.4714 | -0.280176 | -1.69912 | 0.476053 | -0.295051 | -1.62475 | 0.479383 |
| -0.247339 | -1.7633 | 0.436134 | -0.260176 | -1.69912 | 0.44207 | -0.275051 | -1.62475 | 0.446888 |
| -0.227339 | -1.7633 | 0.400868 | -0.240176 | -1.69912 | 0.408088 | -0.255051 | -1.62475 | 0.414393 |
| -0.207339 | -1.7633 | 0.365602 | -0.220176 | -1.69912 | 0.374105 | -0.235051 | -1.62475 | 0.381898 |
| -0.187339 | -1.7633 | 0.330336 | -0.200176 | -1.69912 | 0.340123 | -0.215051 | -1.62475 | 0.349403 |
| -0.167339 | -1.7633 | 0.29507 | -0.180176 | -1.69912 | 0.30614 | -0.195051 | -1.62475 | 0.316908 |
| -0.147339 | -1.7633 | 0.259804 | -0.160176 | -1.69912 | 0.272158 | -0.175051 | -1.62475 | 0.284413 |
| -0.127339 | -1.7633 | 0.224538 | -0.140176 | -1.69912 | 0.238176 | -0.155051 | -1.62475 | 0.251918 |
| -0.107339 | -1.7633 | 0.189272 | -0.120176 | -1.69912 | 0.204193 | -0.135051 | -1.62475 | 0.219423 |
| -0.0873395 | -1.7633 | 0.154006 | -0.100176 | -1.69912 | 0.170211 | -0.115051 | -1.62475 | 0.186928 |
| -0.0673395 | -1.7633 | 0.11874 | -0.0801758 | -1.69912 | 0.136228 | -0.0950506 | -1.62475 | 0.154433 |
| -0.0473395 | -1.7633 | 0.0834738 | -0.0601758 | -1.69912 | 0.102246 | -0.0750506 | -1.62475 | 0.121938 |
| -0.0273395 | -1.7633 | 0.0482078 | -0.0401758 | -1.69912 | 0.0682636 | -0.0550506 | -1.62475 | 0.0894433 |
| -0.0073395 | -1.7633 | 0.0129418 | -0.0201759 | -1.69912 | 0.0342812 | -0.0350506 | -1.62475 | 0.0569484 |
| 0.0126605 | -1.7633 | -0.0223243 | -0.000175869 | -1.69912 | 0.000298823 | -0.0150506 | -1.62475 | 0.0244535 |
| 0.0326605 | -1.7633 | -0.0575903 | 0.0198241 | -1.69912 | -0.0336836 | 0.00494936 | -1.62475 | -0.00804145 |
| 0.0526605 | -1.7633 | -0.0928563 | 0.0398241 | -1.69912 | -0.067666 | 0.0249494 | -1.62475 | -0.0405364 |
| 0.0726605 | -1.7633 | -0.128122 | 0.0598241 | -1.69912 | -0.101648 | 0.0449493 | -1.62475 | -0.0730313 |
| 0.0926605 | -1.7633 | -0.163388 | 0.0798241 | -1.69912 | -0.135631 | 0.0649493 | -1.62475 | -0.105526 |
| 0.11266 | -1.7633 | -0.198654 | 0.0998241 | -1.69912 | -0.169613 | 0.0849493 | -1.62475 | -0.138021 |
| 0.13266 | -1.7633 | -0.23392 | 0.119824 | -1.69912 | -0.203596 | 0.104949 | -1.62475 | -0.170516 |
| 0.15266 | -1.7633 | -0.269186 | 0.139824 | -1.69912 | -0.237578 | 0.124949 | -1.62475 | -0.203011 |
| 0.17266 | -1.7633 | -0.304452 | 0.159824 | -1.69912 | -0.27156 | 0.144949 | -1.62475 | -0.235506 |
| 0.19266 | -1.7633 | -0.339718 | 0.179824 | -1.69912 | -0.305543 | 0.164949 | -1.62475 | -0.268001 |
| 0.21266 | -1.7633 | -0.374984 | 0.199824 | -1.69912 | -0.339525 | 0.184949 | -1.62475 | -0.300496 |
| 0.23266 | -1.7633 | -0.41025 | 0.219824 | -1.69912 | -0.373507 | 0.204949 | -1.62475 | -0.332991 |
| 0.25266 | -1.7633 | -0.445516 | 0.239824 | -1.69912 | -0.40749 | 0.224949 | -1.62475 | -0.365485 |
| 0.27266 | -1.7633 | -0.480782 | 0.259824 | -1.69912 | -0.441472 | 0.244949 | -1.62475 | -0.39798 |
| 0.29266 | -1.7633 | -0.516048 | 0.279824 | -1.69912 | -0.475454 | 0.264949 | -1.62475 | -0.430475 |
| 0.31266 | -1.7633 | -0.551314 | 0.299824 | -1.69912 | -0.509436 | 0.284949 | -1.62475 | -0.46297 |
| 0.33266 | -1.7633 | -0.586579 | 0.319824 | -1.69912 | -0.543418 | 0.304949 | -1.62474 | -0.495464 |
| 0.35266 | -1.7633 | -0.621844 | 0.339823 | -1.69912 | -0.5774 | 0.324949 | -1.62474 | -0.527959 |
| 0.372659 | -1.7633 | -0.657109 | 0.359823 | -1.69912 | -0.611381 | 0.344949 | -1.62474 | -0.560453 |
| 0.392659 | -1.76329 | -0.692372 | 0.379823 | -1.69911 | -0.645362 | 0.364948 | -1.62474 | -0.592947 |
| 0.412658 | -1.76329 | -0.727635 | 0.399822 | -1.69911 | -0.679342 | 0.384948 | -1.62474 | -0.62544 |
| 0.432656 | -1.76328 | -0.762895 | 0.419821 | -1.6991 | -0.71332 | 0.404947 | -1.62474 | -0.657932 |
| 0.452654 | -1.76327 | -0.798151 | 0.439819 | -1.6991 | -0.747296 | 0.424946 | -1.62473 | -0.690422 |
| 0.47265 | -1.76325 | -0.833402 | 0.459817 | -1.69908 | -0.781267 | 0.444944 | -1.62472 | -0.72291 |
| 0.492644 | -1.76322 | -0.868641 | 0.479812 | -1.69906 | -0.815231 | 0.464941 | -1.62471 | -0.755393 |
| 0.512634 | -1.76317 | -0.90386 | 0.499805 | -1.69903 | -0.849183 | 0.484937 | -1.62468 | -0.787868 |
| 0.532615 | -1.76308 | -0.939042 | 0.519793 | -1.69896 | -0.88311 | 0.504929 | -1.62464 | -0.820329 |
| 0.552582 | -1.76291 | -0.974151 | 0.539771 | -1.69885 | -0.916991 | 0.524915 | -1.62457 | -0.852762 |
| 0.572518 | -1.76259 | -1.00911 | 0.55973 | -1.69865 | -0.950784 | 0.54489 | -1.62445 | -0.885145 |
| 0.592395 | -1.76197 | -1.04378 | 0.579652 | -1.69826 | -0.984398 | 0.564843 | -1.62422 | -0.917427 |
| 0.612152 | -1.76076 | -1.07785 | 0.5995 | -1.6975 | -1.01765 | 0.584754 | -1.62377 | -0.949506 |
| 0.631671 | -1.75836 | -1.1107 | 0.6192 | -1.696 | -1.05016 | 0.60458 | -1.6229 | -0.981174 |
| 0.650729 | -1.75365 | -1.14115 | 0.638606 | -1.69303 | -1.08118 | 0.624236 | -1.62118 | -1.012 |
| 0.668952 | -1.74476 | -1.16716 | 0.657453 | -1.68727 | -1.1093 | 0.643557 | -1.61778 | -1.04113 |
| 0.685829 | -1.72915 | -1.1859 | 0.675316 | -1.67658 | -1.13222 | 0.662245 | -1.61122 | -1.06703 |
| 0.70088 | -1.7044 | -1.19458 | 0.691671 | -1.65836 | -1.14704 | 0.679848 | -1.59924 | -1.08724 |
| 0.713873 | -1.66936 | -1.19171 | 0.706092 | -1.63046 | -1.15126 | 0.695842 | -1.57921 | -1.09888 |
| 0.724905 | -1.62453 | -1.17763 | 0.718455 | -1.59227 | -1.14398 | 0.709849 | -1.54925 | -1.09973 |
| 0.734267 | -1.57133 | -1.15378 | 0.728933 | -1.54467 | -1.12596 | 0.721813 | -1.50906 | -1.08926 |
| 0.742288 | -1.51144 | -1.12192 | 0.737845 | -1.48923 | -1.09882 | 0.73195 | -1.45975 | -1.06847 |
| 0.749257 | -1.44628 | -1.08364 | 0.745512 | -1.42756 | -1.06426 | 0.740588 | -1.40294 | -1.039 |
| 0.755401 | -1.37701 | -1.04019 | 0.752204 | -1.36102 | -1.02376 | 0.74804 | -1.3402 | -1.00252 |
| 0.760894 | -1.30447 | -0.992563 | 0.75813 | -1.29065 | -0.978479 | 0.754563 | -1.27282 | -0.960419 |
| 0.765866 | -1.22933 | -0.941501 | 0.763449 | -1.21724 | -0.929304 | 0.760356 | -1.20178 | -0.913782 |
| 0.770416 | -1.15208 | -0.887579 | 0.768281 | -1.1414 | -0.876917 | 0.765569 | -1.12785 | -0.863443 |
| 0.774619 | -1.07309 | -0.831239 | 0.772716 | -1.06358 | -0.821843 | 0.770315 | -1.05157 | -0.810043 |
| 0.778534 | -0.992671 | -0.772828 | 0.776824 | -0.98412 | -0.764488 | 0.774679 | -0.973396 | -0.75407 |
| 0.782208 | -0.91104 | -0.712623 | 0.78066 | -0.9033 | -0.705171 | 0.778729 | -0.893645 | -0.695908 |
| 0.785677 | -0.828385 | -0.650843 | 0.784267 | -0.821334 | -0.644145 | 0.782516 | -0.81258 | -0.635857 |
| 0.78897 | -0.744852 | -0.587666 | 0.787678 | -0.738392 | -0.581616 | 0.786081 | -0.730406 | -0.574158 |
| 0.792113 | -0.660563 | -0.52324 | 0.790923 | -0.654613 | -0.517749 | 0.789457 | -0.647286 | -0.511005 |
| 0.795123 | -0.575616 | -0.457686 | 0.794022 | -0.570111 | -0.45268 | 0.792671 | -0.563354 | -0.446554 |
| 0.798019 | -0.490093 | -0.391103 | 0.796995 | -0.484977 | -0.386524 | 0.795744 | -0.478718 | -0.380936 |
| 0.800812 | -0.404062 | -0.323578 | 0.799858 | -0.39929 | -0.319375 | 0.798693 | -0.393467 | -0.31426 |
| 0.803516 | -0.317581 | -0.255182 | 0.802623 | -0.313114 | -0.251312 | 0.801535 | -0.307677 | -0.246614 |
| 0.80614 | -0.2307 | -0.185976 | 0.805301 | -0.226504 | -0.182404 | 0.804282 | -0.22141 | -0.178076 |
| 0.808692 | -0.143459 | -0.116014 | 0.807901 | -0.139507 | -0.112708 | 0.806944 | -0.134719 | -0.108711 |
| 0.811179 | -0.0558955 | -0.0453413 | 0.810432 | -0.0521622 | -0.042274 | 0.80953 | -0.0476492 | -0.0385735 |
| 0.813608 | 0.0319601 | 0.026003 | 0.812901 | 0.0354951 | 0.028854 | 0.812048 | 0.0397605 | 0.0322874 |
| 0.815984 | 0.12008 | 0.0979837 | 0.815313 | 0.123435 | 0.100639 | 0.814505 | 0.127477 | 0.10383 |
| 0.818312 | 0.208442 | 0.170571 | 0.817673 | 0.211633 | 0.173047 | 0.816906 | 0.215471 | 0.176019 |
| 0.820595 | 0.297024 | 0.243736 | 0.819987 | 0.300065 | 0.246049 | 0.819257 | 0.303717 | 0.248822 |
| 0.822838 | 0.385808 | 0.317457 | 0.822258 | 0.388711 | 0.319621 | 0.821561 | 0.392194 | 0.322211 |
| 0.825045 | 0.474777 | 0.391712 | 0.824489 | 0.477554 | 0.393738 | 0.823824 | 0.480881 | 0.396161 |
| 0.827216 | 0.563918 | 0.466482 | 0.826684 | 0.566579 | 0.468382 | 0.826048 | 0.569761 | 0.47065 |
| 0.829357 | 0.653217 | 0.54175 | 0.828846 | 0.65577 | 0.543532 | 0.828236 | 0.65882 | 0.545658 |
| 0.831467 | 0.742663 | 0.6175 | 0.830977 | 0.745116 | 0.619174 | 0.830391 | 0.748043 | 0.621169 |
| 0.833551 | 0.832246 | 0.693719 | 0.833079 | 0.834605 | 0.695292 | 0.832516 | 0.837419 | 0.697165 |

| 31nm | | 47nm | | 63nm | | 78nm | | 93nm | | 107nm | |
| --- | --- | --- | --- | --- | --- | --- | --- | --- | --- | --- | --- |
| Primary Source Wavelength | External Quantum Efficiency | Primary Source Wavelength | External  Quantum Efficiency | Primary Source Wavelength | External Quantum Efficiency | Primary Source Wavelength | External Quantum Efficiency | Primary Source Wavelength | External Quantum Efficiency | Primary Source Wavelength | External Quantum Efficiency |
| 300 | 76.8303 | 300 | 91.2701 | 300 | 62.2677 | 300 | 44.7758 | 300 | 42.7001 | 300 | 53.3424 |
| 300 | 76.8303 | 300 | 91.2701 | 300 | 62.2677 | 300 | 44.7758 | 300 | 42.7001 | 300 | 53.3424 |
| 310 | 74.4018 | 310 | 93.8255 | 310 | 68.4489 | 310 | 48.4788 | 310 | 43.6257 | 310 | 50.8237 |
| 320 | 72.2896 | 320 | 95.031 | 320 | 74.3354 | 320 | 52.5078 | 320 | 45.0754 | 320 | 49.3276 |
| 330 | 70.7247 | 330 | 95.4016 | 330 | 80.1246 | 330 | 57.2153 | 330 | 47.3579 | 330 | 49.0306 |
| 340 | 69.7394 | 340 | 95.1992 | 340 | 85.5649 | 340 | 62.5575 | 340 | 50.4823 | 340 | 49.8345 |
| 350 | 69.3863 | 350 | 94.6947 | 350 | 90.4129 | 350 | 68.4412 | 350 | 54.4863 | 350 | 51.7208 |
| 360 | 69.7676 | 360 | 94.1486 | 360 | 94.4533 | 360 | 74.6897 | 360 | 59.4211 | 360 | 54.7556 |
| 370 | 71.1005 | 370 | 93.8162 | 370 | 97.4905 | 370 | 80.982 | 370 | 65.323 | 370 | 59.1112 |
| 380 | 73.9489 | 380 | 93.9949 | 380 | 99.2231 | 380 | 86.6747 | 380 | 72.1367 | 380 | 65.1561 |
| 390 | 82.33 | 390 | 95.4345 | 390 | 97.1963 | 390 | 88.6459 | 390 | 79.1438 | 390 | 74.7228 |
| 400 | 84.1443 | 400 | 95.3705 | 400 | 96.4462 | 400 | 88.6954 | 400 | 80.1506 | 400 | 76.0667 |
| 410 | 84.2318 | 410 | 95.1487 | 410 | 96.7558 | 410 | 89.6609 | 410 | 81.1571 | 410 | 76.5569 |
| 420 | 84.2972 | 420 | 94.9417 | 420 | 97.0329 | 420 | 90.5719 | 420 | 82.1506 | 420 | 77.0983 |
| 430 | 84.3253 | 430 | 94.7295 | 430 | 97.2708 | 430 | 91.4317 | 430 | 83.1347 | 430 | 77.6887 |
| 440 | 84.267 | 440 | 94.4534 | 440 | 97.4176 | 440 | 92.1985 | 440 | 84.0724 | 440 | 78.2911 |
| 450 | 83.9684 | 450 | 93.937 | 450 | 97.2953 | 450 | 92.7064 | 450 | 84.8135 | 450 | 78.765 |
| 460 | 83.1335 | 460 | 92.8463 | 460 | 96.5566 | 460 | 92.6204 | 460 | 85.0492 | 460 | 78.8252 |
| 470 | 81.5139 | 470 | 90.9023 | 470 | 94.9046 | 470 | 91.6436 | 470 | 84.5 | 470 | 78.2144 |
| 480 | 79.3106 | 480 | 88.3279 | 480 | 92.5632 | 480 | 89.9762 | 480 | 83.3393 | 480 | 77.0936 |
| 490 | 77.2315 | 490 | 85.9076 | 490 | 90.3492 | 490 | 88.4019 | 490 | 82.2843 | 490 | 76.1236 |
| 500 | 75.4684 | 500 | 83.8428 | 500 | 88.4662 | 500 | 87.1119 | 500 | 81.5068 | 500 | 75.4612 |
| 510 | 73.7949 | 510 | 81.8751 | 510 | 86.6391 | 510 | 85.8309 | 510 | 80.7424 | 510 | 74.8564 |
| 520 | 72.0532 | 520 | 79.8288 | 520 | 84.684 | 520 | 84.3735 | 520 | 79.8083 | 520 | 74.1326 |
| 530 | 70.2101 | 530 | 77.6697 | 530 | 82.5684 | 530 | 82.7055 | 530 | 78.6632 | 530 | 73.2436 |
| 540 | 68.2741 | 540 | 75.4092 | 540 | 80.3088 | 540 | 80.8426 | 540 | 77.3145 | 540 | 72.1879 |
| 550 | 66.2551 | 550 | 73.0604 | 550 | 77.9232 | 550 | 78.8032 | 550 | 75.7731 | 550 | 70.9683 |
| 560 | 64.1637 | 560 | 70.6369 | 560 | 75.4301 | 560 | 76.607 | 560 | 74.0529 | 560 | 69.5904 |
| 570 | 62.0115 | 570 | 68.1526 | 570 | 72.8479 | 570 | 74.2748 | 570 | 72.1694 | 570 | 68.0621 |
| 580 | 59.8097 | 580 | 65.6211 | 580 | 70.1948 | 580 | 71.8271 | 580 | 70.1398 | 580 | 66.3938 |
| 590 | 57.5701 | 590 | 63.0561 | 590 | 67.4886 | 590 | 69.2848 | 590 | 67.9825 | 590 | 64.5974 |
| 600 | 55.3038 | 600 | 60.4705 | 600 | 64.746 | 600 | 66.668 | 600 | 65.716 | 600 | 62.6862 |
| 610 | 53.0219 | 610 | 57.877 | 610 | 61.983 | 610 | 63.9961 | 610 | 63.3591 | 610 | 60.6744 |
| 620 | 50.735 | 620 | 55.2871 | 620 | 59.2144 | 620 | 61.2875 | 620 | 60.9306 | 620 | 58.5766 |
| 630 | 48.4528 | 630 | 52.7118 | 630 | 56.4541 | 630 | 58.5596 | 630 | 58.4483 | 630 | 56.4079 |
| 640 | 46.1846 | 640 | 50.1611 | 640 | 53.7145 | 640 | 55.8281 | 640 | 55.9295 | 640 | 54.1833 |
| 650 | 43.9389 | 650 | 47.6442 | 650 | 51.007 | 650 | 53.1077 | 650 | 53.3906 | 650 | 51.9174 |
| 660 | 41.7235 | 660 | 45.1692 | 660 | 48.3415 | 660 | 50.4115 | 660 | 50.8466 | 660 | 49.6244 |
| 670 | 39.5453 | 670 | 42.7434 | 670 | 45.727 | 670 | 47.7512 | 670 | 48.3114 | 670 | 47.3177 |
| 680 | 37.4105 | 680 | 40.3731 | 680 | 43.1712 | 680 | 45.1372 | 680 | 45.7977 | 680 | 45.01 |
| 690 | 35.3246 | 690 | 38.0638 | 690 | 40.6806 | 690 | 42.5785 | 690 | 43.3168 | 690 | 42.713 |
| 700 | 33.2922 | 700 | 35.8201 | 700 | 38.2608 | 700 | 40.0827 | 700 | 40.8788 | 700 | 40.4375 |
| 710 | 31.3174 | 710 | 33.6459 | 710 | 35.9165 | 710 | 37.6564 | 710 | 38.4924 | 710 | 38.1932 |
| 720 | 29.4034 | 720 | 31.5443 | 720 | 33.6512 | 720 | 35.3049 | 720 | 36.1652 | 720 | 35.989 |
| 730 | 27.553 | 730 | 29.5177 | 730 | 31.4679 | 730 | 33.0326 | 730 | 33.9037 | 730 | 33.8324 |
| 740 | 25.7684 | 740 | 27.5679 | 740 | 29.3686 | 740 | 30.8429 | 740 | 31.7131 | 740 | 31.7303 |
| 750 | 24.0511 | 750 | 25.6961 | 750 | 27.3547 | 750 | 28.7383 | 750 | 29.5978 | 750 | 29.6882 |
| 760 | 22.4023 | 760 | 23.9031 | 760 | 25.427 | 760 | 26.7205 | 760 | 27.5611 | 760 | 27.7111 |
| 770 | 20.8225 | 770 | 22.189 | 770 | 23.5859 | 770 | 24.7907 | 770 | 25.6056 | 770 | 25.8028 |
| 780 | 19.3122 | 780 | 20.5538 | 780 | 21.831 | 780 | 22.9491 | 780 | 23.733 | 780 | 23.9665 |
| 790 | 17.8713 | 790 | 18.997 | 790 | 20.1618 | 790 | 21.1957 | 790 | 21.9445 | 790 | 22.2047 |
| 800 | 16.4992 | 800 | 17.5176 | 800 | 18.5772 | 800 | 19.53 | 800 | 20.2406 | 800 | 20.5189 |
| 810 | 15.1955 | 810 | 16.1146 | 810 | 17.0759 | 810 | 17.9509 | 810 | 18.6211 | 810 | 18.9103 |
| 820 | 13.9592 | 820 | 14.7867 | 820 | 15.6565 | 820 | 16.4571 | 820 | 17.0856 | 820 | 17.3795 |
| 830 | 12.910 | 830 | 13.5322 | 830 | 14.317 | 830 | 15.047 | 830 | 15.6331 | 830 | 15.9264 |
| 840 | 11.684 | 840 | 12.960 | 840 | 13.0555 | 840 | 13.7188 | 840 | 14.2623 | 840 | 14.5507 |
| 850 | 10.250 | 850 | 11.680 | 850 | 11.990 | 850 | 12.030 | 850 | 12.170 | 850 | 13.2517 |
| 860 | 9.66293 | 860 | 10.210 | 860 | 11.790 | 860 | 11.930 | 860 | 11.950 | 860 | 12.0282 |
| 870 | 8.74373 | 870 | 9.21332 | 870 | 9.71732 | 870 | 10.350 | 870 | 10.360 | 870 | 10.880 |
| 880 | 7.88314 | 880 | 8.29837 | 880 | 8.74563 | 880 | 9.18046 | 880 | 9.56192 | 880 | 9.80204 |
| 890 | 7.07935 | 890 | 7.44513 | 890 | 7.84045 | 890 | 8.22763 | 890 | 8.57212 | 890 | 8.79601 |
| 900 | 6.33052 | 900 | 6.65141 | 900 | 6.99933 | 900 | 7.34246 | 900 | 7.65181 | 900 | 7.85877 |
| 910 | 5.63478 | 910 | 5.91502 | 910 | 6.21981 | 910 | 6.52237 | 910 | 6.79854 | 910 | 6.98822 |
| 920 | 4.99024 | 920 | 5.23379 | 920 | 5.49944 | 920 | 5.76479 | 920 | 6.00982 | 920 | 6.18219 |
| 930 | 4.39502 | 930 | 4.60553 | 930 | 4.83578 | 930 | 5.06714 | 930 | 5.28311 | 930 | 5.43842 |
| 940 | 3.84722 | 940 | 4.0281 | 940 | 4.22645 | 940 | 4.42688 | 940 | 4.61589 | 940 | 4.460 |
| 950 | 3.34498 | 950 | 3.49936 | 950 | 3.66908 | 950 | 3.84149 | 950 | 4.00565 | 950 | 4.1284 |
| 960 | 2.88643 | 960 | 3.01722 | 960 | 3.16136 | 960 | 3.30852 | 960 | 3.44991 | 960 | 3.55748 |
| 970 | 2.46975 | 970 | 2.57964 | 970 | 2.70102 | 970 | 2.82554 | 970 | 2.620 | 970 | 3.03949 |
| 980 | 2.09316 | 980 | 2.18462 | 980 | 2.28587 | 980 | 2.39021 | 980 | 2.49213 | 980 | 2.57213 |
| 990 | 1.75489 | 990 | 1.020 | 990 | 1.91375 | 990 | 2.00023 | 990 | 2.08534 | 990 | 2.15309 |
| 1000 | 1.45323 | 1000 | 1.450 | 1000 | 1.260 | 1000 | 1.65337 | 1000 | 1.72354 | 1000 | 1.78012 |
| 1010 | 1.18652 | 1010 | 1.23566 | 1010 | 1.29039 | 1010 | 1.34749 | 1010 | 1.40449 | 1010 | 1.45102 |
| 1020 | 0.953118 | 1020 | 0.991903 | 1020 | 1.03518 | 1020 | 1.0805 | 1020 | 1.12604 | 1020 | 1.16364 |
| 1030 | 0.751457 | 1030 | 0.781506 | 1030 | 0.815093 | 1030 | 0.850397 | 1030 | 0.886083 | 1030 | 0.915869 |
| 1040 | 0.580006 | 1040 | 0.602801 | 1040 | 0.628321 | 1040 | 0.655239 | 1040 | 0.682607 | 1040 | 0.705683 |
| 1050 | 0.437288 | 1050 | 0.45418 | 1050 | 0.473123 | 1050 | 0.493168 | 1050 | 0.513662 | 1050 | 0.531109 |
| 1060 | 0.321874 | 1060 | 0.334096 | 1060 | 0.347824 | 1060 | 0.362397 | 1060 | 0.377375 | 1060 | 0.39024 |
| 1070 | 0.230552 | 1070 | 0.23916 | 1070 | 0.248841 | 1070 | 0.25915 | 1070 | 0.269798 | 1070 | 0.279023 |
| 1080 | 0.157733 | 1080 | 0.163524 | 1080 | 0.170046 | 1080 | 0.17701 | 1080 | 0.184239 | 1080 | 0.190552 |
| 1090 | 0.102281 | 1090 | 0.105974 | 1090 | 0.110138 | 1090 | 0.114598 | 1090 | 0.119248 | 1090 | 0.12334 |
| 1100 | 0.0633021 | 1100 | 0.0655495 | 1100 | 0.0680879 | 1100 | 0.0708132 | 1100 | 0.0736671 | 1100 | 0.0761968 |
| 1110 | 0.0394377 | 1110 | 0.0408149 | 1110 | 0.0423724 | 1110 | 0.0440488 | 1110 | 0.0458116 | 1110 | 0.047385 |
| 1120 | 0.0232088 | 1120 | 0.024006 | 1120 | 0.0249088 | 1120 | 0.0258829 | 1120 | 0.0269112 | 1120 | 0.027835 |
| 1130 | 0.0121422 | 1130 | 0.0125526 | 1130 | 0.0130178 | 1130 | 0.0135209 | 1130 | 0.0140541 | 1130 | 0.0145361 |
| 1140 | 0.00597133 | 1140 | 0.00616689 | 1140 | 0.00639528 | 1140 | 0.00663956 | 1140 | 0.00689938 | 1140 | 0.00713563 |
| 1150 | 0.00372034 | 1150 | 0.00384209 | 1150 | 0.00398042 | 1150 | 0.00413067 | 1150 | 0.004246 | 1150 | 0.00443769 |
| 1160 | 0.00222604 | 1160 | 0.00229774 | 1160 | 0.00237928 | 1160 | 0.00246563 | 1160 | 0.00256309 | 1160 | 0.00264788 |
| 1170 | 0.00112676 | 1170 | 0.00116248 | 1170 | 0.00120609 | 1170 | 0.00123681 | 1170 | 0.00129832 | 1170 | 0.0013418 |
| 1180 | 0.000410282 | 1180 | 0.000422465 | 1180 | 0.000437462 | 1180 | 0.000454285 | 1180 | 0.000471492 | 1180 | 0.000481875 |
| 1190 | 5.64806e-005 | 1190 | 5.86591e-005 | 1190 | 6.00613e-005 | 1190 | 5.64806e-005 | 1190 | 5.87E-05 | 1190 | 6.00613e-005 |
| 1200 | 7.46249e-012 | 1200 | 7.75036e-012 | 1200 | 7.93559e-012 | 1200 | 7.46249e-012 | 1200 | 7.75E-12 | 1200 | 7.93559e-012 |
|  |  |  |  |  |  |  |  |  |  |  |  |

**ARC ZnO layer thickness calculation**

| wavelength | RI | Thickness (nm) | Isc | Voc | Eff |
| --- | --- | --- | --- | --- | --- |
| 300 | 2.400 | 31 | 1.552 | 0.807 | 10.270 |
| 400 | 2.100 | 47 | 1.686 | 0.809 | 11.390 |
| 500 | 1.960 | 63 | 1.771 | 0.810 | 11.980 |
| 600 | 1.910 | 78 | 1.763 | 0.810 | 11.910 |
| 700 | 1.880 | 93 | 1.699 | 0.809 | 11.480 |
| 800 | 1.860 | 107 | 1.625 | 0.808 | 10.970 |
